# Motivations for Nonuse as Predictors of Substance Use Among Reservation-Based Youth in a Computerized Screening and Brief Intervention: The Power of Positive People

**DOI:** 10.1080/29973368.2026.2667173

**Published:** 2026-05-07

**Authors:** Caroline M. Barry, Melvin D. Livingston, Eugena Atkinson, Juli R. Skinner, Kelli A. Komro

**Affiliations:** a Emory University, Atlanta, GA, USA; b Cherokee Nation Behavioral Health, Tahlequah, OK, USA

**Keywords:** motivations, protective factors, rural and American Indian adolescents, Screening and Brief Intervention

## Abstract

Adolescent substance use is a critical public health issue in rural and American Indian communities with disparities persisting into young adulthood. This study examines motivations for nonuse as predictors of substance use trajectories among adolescents in a computerized Screening and Brief Intervention in 10 high schools on and near the Cherokee Nation Reservation. Low-risk students (n = 360) reported their motivations for nonuse during the initial Screening and Brief Intervention session, and substance use outcomes (alcohol, cannabis, prescription opioid misuse, vaping) were assessed in subsequent surveys. Results from longitudinal growth curve models indicated that most motivations for nonuse were not associated with differential trajectories of substance use over time. One notable exception was “having positive people in one’s life,” which predicted a significantly lower likelihood of nicotine vaping at the initial follow-up, followed by a faster increase in vaping over subsequent semesters. In secondary wave-specific models, this motivation also predicted reduced cannabis use at the first follow-up, whereas endorsing “it makes me feel bad” predicted higher cannabis use. Reinforcing positive social influences and addressing negative feelings may be an important part of prevention strategies like Screening and Brief Intervention. This study offers new evidence on motivations for nonuse to inform the design of digital prevention tools for underserved rural youth.

Adolescent substance use is a critical public health concern with a well-documented developmental trajectory and a disproportionate burden among rural and American Indian (AI) youth (CDC, 2024; Chen & Jacobson, 2012; Friedman et al., 2022, 2023; Friedman & Hansen, 2022; Prazak et al., 2025). Substance use typically increases from mid to late adolescence, peaks in young adulthood, and declines thereafter (Chen & Jacobson, 2012). Early initiation is associated with higher risk for substance use disorders and long-term harms, including severe consequences among AI youth (Dawson et al., 2008; King & Chassin, 2007; Substance Abuse and Mental Health Services Administration, 2014). For example, alcohol intoxication before age 14 among urban AI adolescents predicted heavier later use and a threefold increase in risk for alcohol use disorder (Henry et al., 2011). Early alcohol and cannabis use onset among reservation-based youth predicted subsequent prescription drug misuse (Swaim & Stanley, 2020). Recent increases in overdose deaths indicate the pressing need for prevention strategies in communities with the greatest burden (Friedman et al., 2022, 2023; Friedman & Hansen, 2022; Prazak et al., 2025).

Most prevention research has examined why adolescents *initiate* and *escalate* use. Far less is known about why they *choose not to use substances*. A strengths-based focus on motivations for nonuse may reveal protective processes and inform culturally grounded prevention strategies (Allen et al., 2022; Henson et al., 2017). Motivations for use and nonuse are not simply opposites but reflect distinct psychological processes (Anderson et al., 2011; Richetin et al., 2011). Prior work documents reasons for not using–such as health concerns, disinterest, fear of consequences, or family expectations (McIntosh et al., 2005; Patrick et al., 2010; Rosansky & Rosenberg, 2019), but research has been largely atheoretical.

Self-Determination Theory (SDT) provides a helpful framework for understanding motivations for nonuse. SDT conceptualizes motivation along a continuum from controlled regulation (e.g., avoiding punishment, meeting others’ expectations) to more autonomous regulation (e.g., valuing health, integrating abstinence with one’s identity; Ryan & Deci, 2017). Autonomous motivations predict healthier and more sustained behavioral outcomes, while controlled motivations have shown to be less protective or even maladaptive (Li et al., 2013; Ng et al., 2012; Owen et al., 2014; Teixeira et al., 2012). Autonomous motivations are believed to predict higher quality of life and maintenance of healthier behaviors due to deeper internalization (Koestner et al., 2010; Li et al., 2013; Moore & Hardy, 2020). Across 73 studies included in a 2021 meta-analysis, SDT-informed interventions showed small-to-medium positive effects on SDT constructs and a range of health outcomes. Positive changes in autonomous motivation—but not controlled motivation or lack of motivation altogether—were consistently associated with positive changes in health behavior (Ntoumanis et al., 2021).

Research on SDT applied to abstinence from substance use is sparse. In one study of 475 adolescents aged 16–19, Moore and Hardy (2020) found that more autonomous motivations for abstinence predicted reduced substance use (e.g., avoiding drinking, drugs, and smoking “because I will feel bad about myself if I do any of those things” or “because I think it’s important to avoid those behaviors), whereas controlled motivations (e.g., avoiding drinking, drugs, and smoking “because I will get in trouble if I do any of those things” or “because my parents will be disappointed”) predicted stable or increased use. This distinction indicates the importance of examining the type of motivation rather than whether youth report nonuse motivations at all.

Guided by SDT, this study examined whether motivations for nonuse predicted trajectories of substance use among adolescents living on or near the Cherokee Nation Reservation. We focused on “low-risk” youth (i.e., those with low to no substance use at baseline; n = 360) and three follow-up waves of survey data on their alcohol use, cannabis use, prescription opioid misuse, and vaping. In line with SDT and prior evidence, we hypothesized that more autonomous motivations (e.g., wanting to be healthy, focusing on the future) would be associated with slower increases in substance use over time, whereas more controlled motivations (e.g., avoiding getting in trouble) would show weaker or no protective effects. As a secondary aim, we examined whether baseline motivations predicted substance use at the initial follow-up.

## Materials and methods

### Theoretical framework

**Figure 1.**
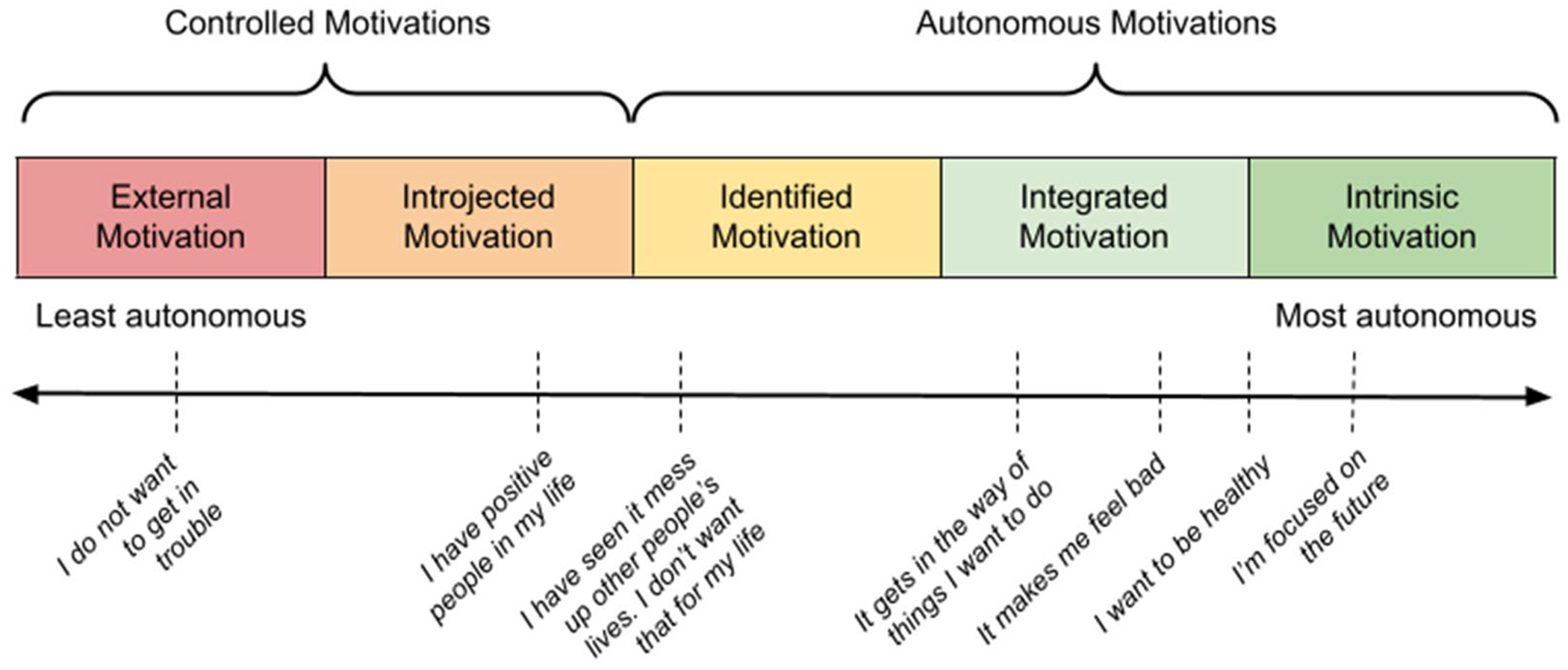
Self-determination theory spectrum of motivations for nonuse. Adapted from Moore and Hardy (2020) to include motivations from the computerized Screening and Brief Intervention along the spectrum.

SDT describes motivations in terms of autonomy, defined as the “need to perceive one’s actions as authentic and volitional” (Moore & Hardy, 2020; Ryan & Deci, 2017). Motivations can be positioned on a spectrum of autonomy from extrinsic to intrinsic (Figure 1). At the controlled end, behaviors are driven by external pressures or punishments and rewards, such as not using substances to avoid getting in trouble (Moore & Hardy, 2020; Ryan & Deci, 2017). More internalized forms include introjected motivation (i.e., acting to maintain self-esteem or avoid shame) and identified motivation (i.e., recognizing the personal importance of a behavior). Some motivations assessed in this study, such as having positive people in one’s life or viewing substance use as an obstacle, could reflect these mid-range forms of motivation. At the autonomous end, integrated and intrinsic motivations align with one’s values or goals and fuel purpose or contentment (e.g., focusing on the future or wanting to be healthy) (Moore & Hardy, 2020).

### Computerized Screening and Brief Intervention

Baseline motivations for nonuse were collected via a computerized Screening and Brief Intervention (SBI) program implemented in high schools randomized to the intervention arm of the Cherokee Nation prevention trial (Komro et al., 2022, 2025). SBI strategies are used to (1) assess for problem levels of use, (2) deliver a commensurate dose of short but powerful interactive exercises that operate on motivations to change, and (3) follow up with individuals who report monthly use for an in-person motivational interviewing (MI) session. Brief interventions have demonstrated effectiveness for reducing substance misuse (Steele et al., 2020).

The computerized SBI draws on MI, which is suited to be culturally and developmentally appropriate for students in this context (Garrett et al., 2019). MI aligns with values common to many American Indian communities, such as respect, collaboration, and non-confrontation (Dickerson et al., 2016), and MI supports adolescents’ growing need for autonomy (Li et al., 2016).

### Setting and sample

We analyzed self-reported motivations for nonuse among adolescents who screened as low-risk (i.e., low to no alcohol use, cannabis use, prescription opioid misuse, and vaping). Motivations (primary predictors) were drawn from the computerized SBI, and covariates and outcomes were drawn from the trial’s surveys.

Data were collected through the Cherokee Nation multilevel prevention trial in high schools. Detailed school- and student-level eligibility criteria are reported in the trial protocol paper (Komro et al., 2022). Parental consent was obtained by mail with follow-up by telephone or home visit as needed. Students provided informed assent (or consent if aged 18 years or older) and were free to withdraw at any time. The analytic sample included students from 10 intervention schools, 8 of which were located within the boundaries of the Cherokee Nation Reservation and 2 within a neighboring Tribal Nation’s reservation. Consistent with prior publications and Cherokee Nation IRB guidance, which acts as the institutional review board with an agreement letter from the neighboring Tribal Nation, we refer to students as attending schools “on or near” the Cherokee Nation Reservation. Students completed the computerized SBI–with guided MI and response-based branching–twice annually. Motivations for nonuse were collected during the first SBI session (Spring 2022) before any motivational interviewing. Substance use outcomes come from waves 3–5 (Fall 2022, Spring 2023, and Fall 2023). Covariates were obtained at wave 1 (Fall 2021). Wave 2 (Spring 2022) survey data were excluded due to inconsistent timing relative to computerized SBI administration across schools during this semester. Some schools had surveys before computerized SBI, and others after. This variability made wave 2 survey data unsuitable for longitudinal modeling.

The analytic sample included students who reported low to no use (“never” or “once or twice”) of alcohol, cannabis, prescription opioids, and vapes in the past six months during computerized screening and completed subsequent questions about their motivations for nonuse. Of the 433 students who completed screening, 360 (83%) screened as low risk across all substances and completed the motivations-for-nonuse items. Students who reported monthly or weekly use of any substance were branched into motivational interviewing and excluded from analyses.

### Measures

#### Motivations for nonuse

Motivations for nonuse were assessed after screening in the computerized SBI. It asked, “What is the most important reason you have not used?” Respondents marked “yes” or “no” (1 or 0) to the following statements: “I have positive people in my life,” “I do not want to get in trouble,” “It gets in the way of things I want to do,” “I’m focused on the future,” “I want to be healthy,” “I have seen it mess up other people’s lives. I don’t want that for my life,” and “It makes me feel bad.” Items were developed for the computerized SBI and have not been psychometrically validated. They were intended as brief, face-valid prompts to elicit reasons for nonuse. As such, SDT was retroactively applied to this secondary data source for the present study aims.

#### Substance use

Alcohol use, cannabis use, prescription opioid misuse, and vaping were assessed on the survey. Students were asked, “During the past 30 days, how many days did you drink alcohol?”, “…how many days did you use marijuana?”, “…how many days did you use a prescription opioid without a doctor’s prescription or differently than how a doctor or medical provider told you to use it?”, and “…how many days have you vaped nicotine?” Responses to each item were reported as an integer from 0 to 30 days.

#### Covariates

All motivations were included in the models, with the focal motivation incorporated into the interaction term and rotated through to test each one in separate models (see Analytic approach). Additional covariates came from the baseline wave of the survey (Fall 2021). These included age, gender, enrollment in a free or reduced-price lunch program at school (an indicator of household socioeconomic status), race/ethnicity, and baseline use of the substance specific to each model. Covariates were selected based on theory and empirical evidence suggesting that motivations and substance use differ by age, gender, race/ethnicity, and socioeconomic status, and that substance use at one time point strongly predicts future use (Chambers et al., 2003; Henry et al., 2011; Komro et al., 2016, 2022; Laudet & Stanick, 2010; Moore & Hardy, 2020; Patrick et al., 2012; Ryan & Deci, 2017; Smith et al., 2017; Stanley et al., 2020; Swaim & Stanley, 2020).

#### Analytic approach

Data were analyzed using SAS 9.4 (SAS Institute Inc, 2013). Univariate analyses were conducted to assess frequencies, distributions, and missingness, followed by bivariate correlations for continuous variables and chi-square tests for categorical variables, including binary past-month substance use outcomes.

To examine whether baseline motivations for nonuse predicted changes in substance use over time, we fit longitudinal mixed-effects Poisson regression models including repeated measures from waves 3–5. Time was coded such that wave 3 = 0, wave 4 = 1, and wave 5 = 2. Each model included the focal motivation for nonuse, time, their interaction, and baseline covariates. A random intercept for school accounted for clustering of students within schools. Within-student correlation across repeated measures was modeled using a compound-symmetry residual covariance structure. Because only three repeated outcome measurements were available per participant and outcomes exhibited floor effects, random slopes for time were not estimated to avoid unstable or non-identifiable variance components (Luke, 2020; Singer & Willett, 2003). Our models focused on mean trajectories rather than individual variability in change.

Separate models were estimated for each of the seven baseline motivations across four substance use outcomes (28 total models), consistent with prior SDT research conceptualizing each motivation as a distinct construct (e.g., Moore & Hardy, 2020). The primary parameter of interest was the motivation x time interaction, which tested whether substance use trajectories differed over time between students who did versus did not endorse each motivation. A representative model including fixed effects, the school-level random intercept, and residual covariance parameters from a single imputation is presented in Supplemental Table S0. As a secondary analysis, we estimated separate mixed-effects Poisson models predicting wave 3 outcomes only (the first follow-up) to evaluate initial prospective associations between baseline motivations and subsequent substance use. These models dropped the time term and its interaction. All models assumed a log link and quasi-Poisson variance function. To resolve convergence issues related to modeling constraints while preserving theoretical integrity, one imputation was omitted from the alcohol and vaping models, and three from the cannabis models. Sensitivity analyses with time as a categorical variable were performed with no meaningful change in results.

Missing data were handled using multiple imputation (m = 30) in R (v4.3.1) with the *mice* package. Retention was 85.8% at wave 3, 82.8% at wave 4, and 78.3% at wave 5. Missingness ranged from 6 to 7% for covariates to 13–23% for outcomes across waves 3–5. The multivariate imputation by chained equations (MICE) algorithm was used under a missing-at-random assumption, i.e., missing data depended on qualities of the observed data used to impute missing values (Baraldi & Enders, 2010; Rubin, 1976). Fixed-effect estimates were pooled across imputations in SAS (PROC MIANALYZE).

## Results

Univariate statistics on sample characteristics are shown in Table 1. Among students (n = 360), 54% were 15 years old, 42% were 16, and 4% were older than 16. Half identified as female (50%). Students reported their race/ethnicity as American Indian only (26%), White only (38%), American Indian and at least one other race (23%), or another racial identity (13%). A majority (68%) reported receiving free or reduced-price lunch at school. Across waves, mean past 30-day alcohol use ranged from 0.26 to 0.90 days, cannabis use ranged from 0.42 to 1.56 days, prescription opioid misuse ranged from 0.14 to 0.35 days, and vaping ranged from 1.10 to 1.68 days.

**Figure 2.**
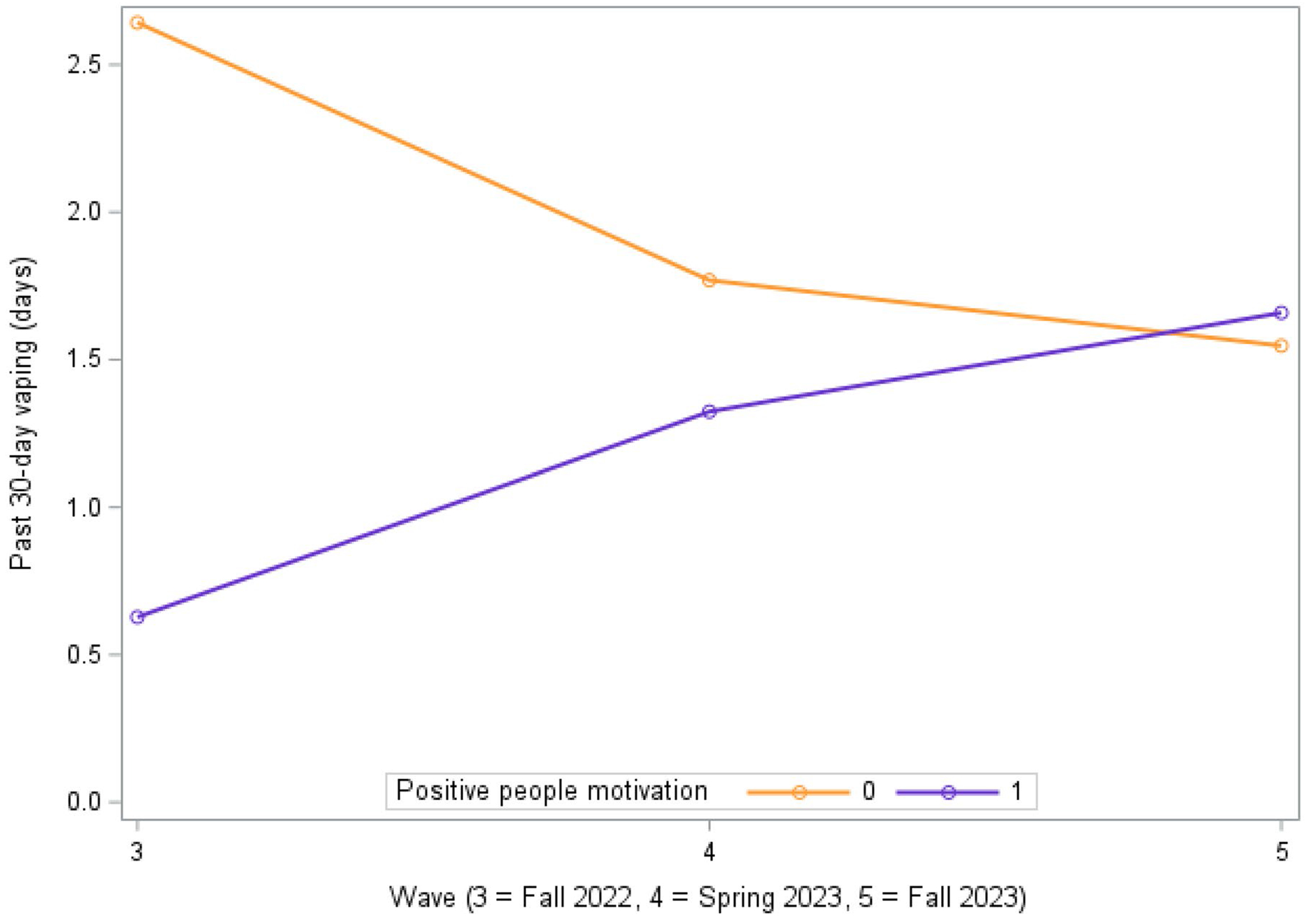
Trajectories of vaping over time by the motivation, “I have positive people in my life”.

Table 2 shows results from the adjusted longitudinal growth curve models with interaction terms testing whether motivations for nonuse predicted changes in substance use trajectories over time. A significant interaction was observed between time and “I have positive people in my life” on vaping. At initial follow-up (wave 3), having positive people was associated with a lower likelihood of vaping (β=−1.409, SE = 0.530, p = 0.008). However, over time, this protective effect diminished, as evidenced by a faster increase in vaping rates for those with positive people compared to those without (β = 0.796, SE = 0.276, p = 0.004; see Figure 2). No other significant trajectory differences were observed. Pooled parameter estimates for all 28 models are provided in Supplemental Tables S1–S28.

Table 3 shows results from adjusted main effects models on initial follow-up (wave 3) outcomes only. In addition to reduced vaping as previously described, “I have positive people in my life” significantly predicted reduced cannabis use (β=−1.847, SE = 0.670, p = 0.008) at initial follow-up, demonstrating protective effects. Conversely, “It makes me feel bad” significantly predicted higher cannabis use (β = 1.226, SE = 0.596, p = 0.039). The remaining models showed no significant main effects on initial follow-up outcomes.

## Discussion

This study examined whether motivations for nonuse predicted trajectories of substance use over time among adolescents living on or near the Cherokee Nation Reservation. The majority of results were not significant. One notable exception was the significant motivation x time interaction showing that “I have positive people in my life” predicted an initially protective effect against vaping that diminished over time. Although both “positive people in my life” and “it makes me feel bad” significantly predicted cannabis use at initial follow-up (wave 3), effects did not translate into differential trajectories over subsequent waves. This pattern suggests that baseline motivations may exert their strongest influence shortly after assessment—capturing proximal influences or momentary protective or vulnerability factors— without necessarily shaping longer-term patterns of cannabis use. Early motivational processes that shape immediate behavior may be overshadowed by evolving social contexts, access, or normative shifts over time.

Regarding the constructs themselves, we originally considered “I have positive people in my life” as a mid-range motivation (i.e., closer to introjected or identified), where adolescents may abstain from substances in part to avoid disappointing positive people in their lives like caregivers or non-using friends, similar to findings by Moore and Hardy (2020); however emulating values of positive people could push its classification more toward the autonomous end of the spectrum, as our results suggest. Endorsing this motivation may indicate gratitude or a healthy outlook on life that positively predicts health behaviors and wellbeing. In addition to the findings on vaping, the main effect of positive people on cannabis use at follow-up was protective, consistent with our theory and the vaping results. Unlike a generic measure of social support, this motivation is explicitly tied to substance use outcomes, making it particularly valuable for motivational interviewing. Further, in rural and Indigenous contexts, positive relationships often reflect relational responsibility and connectedness, which function as protective factors beyond individual volition (Cross, 2007; Hill, 2006; Yamane & Helm, 2022). This points to the need for prevention frameworks that integrate relational worldviews alongside SDT to more fully capture culturally resonant motivations for abstinence.

Endorsing “It makes me feel bad” was significantly predictive of higher cannabis use at initial follow-up. While this may seem initially contradictory to Moore & Hardy’s findings, our measure of “feeling bad” was slightly different from theirs. Moore & Hardy’s item captured anticipated negative feelings about oneself as a consequence of substance use: “I will feel bad about myself if I do any of those things,” which is an integrated motivation with high autonomy. Our item, however, measured feeling bad more generally (e.g., physically or otherwise), not necessarily related to self-image. This difference may be attributed to a construct difference or measurement noise, as our measure might capture aspects of cannabis use that were not accounted for by the baseline use covariate. It could also reflect increased proximity to or greater knowledge of cannabis, thereby increasing salience of adverse consequences. For the remaining models that yielded non-significant findings, it is important to consider that many environmental, social, and individual factors can influence motivations and substance use behaviors–despite accounting for covariates.

This study has several strengths. The research question was theory-driven and intended to directly inform computer-based intervention development and motivational interviewing for adolescents. Motivations span the spectrum of SDT, facilitating the opportunity to assess conceptually distinct levels of motivational autonomy. Additionally, multiple waves of longitudinal outcome data provide a foundation for inference of causal effects. Finally, rural and AI youth are understudied yet shoulder an inequitable burden of drug-related harms, and this research builds on the small but growing body of evidence in partnership with community.

This study was not without limitations. Reliance on secondary data presented inherent constraints. First, sample restriction to the trial’s intervention group was necessitated by the predictors under study; only the intervention group received questions about motivations for nonuse via the computer-based intervention. Also, baseline motivations were assessed prior to exposure to motivational interviewing, but it is possible that some adolescents later escalated their use and received motivational interviewing during follow-ups. Such exposure may have flattened developmental trajectories of use over time, restricting variability in later outcomes. While this limits the ability to isolate predictors from intervention effects, it reflects real-world prevention conditions, where supports are available to youth as their risk increases. Sample restriction also reduced the sample size, which limited power for stratification by subgroup (e.g., AI identity or gender). Additionally, repeated measurements of the motivations for nonuse were not incorporated into analyses due to intervention branching logic; at each time point, students were screened and routed to receive motivation questions accordingly, which would have changed the sample over time. Further, multiple imputation assumes data were missing at random; if this assumption was not met, bias could remain. Additionally, because 28 theory-driven models were estimated, risk of type I error exists. Each motivation reflects a distinct SDT construct, but findings should be interpreted with some caution.

In AI communities, motivations for nonuse are shaped not only by individual decision-making but also by broader cultural influences of connectedness, relational responsibility, and holistic wellbeing. The Relational Worldview model, as an example, emphasizes balance across mind, body, spirit, and context as essential to health (Cross, 2007), while other scholarship proposes how belonging, positive social connections, and harmony with community and culture underpin Indigenous conceptualizations of wellness (Hill, 2006; Yamane & Helm, 2022). Future prevention research in Tribal contexts would benefit from integrating Indigenous models of health alongside SDT to better understand how culturally resonant motivations for nonuse function as levers for intervention.

Other limitations of the secondary data set pertain to measurement and timing. Measures of motivations were built into the computerized SBI but could be stronger as Likert-type items to capture granularity of motivational influences. These measures were not psychometrically tested for validity and reliability. In addition, “low to no use” was defined as reporting “never” or “once or twice” use in the past six months, which does not capture lifetime use. Some adolescents categorized as “low to no use” may therefore have had earlier substance use beyond that window. However, we controlled for substance use reported at wave 1 (six months prior), which mitigates—though does not fully eliminate—this limitation. Further, the timing of baseline data collection may not have been optimal, potentially missing a critical developmental window for substance use escalation among peers excluded from analyses. It is possible that adolescents who report low to no use by 10th grade are predisposed to lower levels of substance use throughout adolescence. Had baseline data collection occurred earlier, e.g., during middle school, significant differences might have been observed on substance use outcomes over time among a larger, more heterogeneous sample. Future studies should consider survey measurement in observational cohorts at an earlier age to mitigate these limitations.

In conclusion, this study offers insights to motivations for nonuse among adolescents in rural and AI communities, specifically within the context of the Cherokee Nation Reservation. The protective effects of positive influences and the unexpected risks associated with certain motivations highlight the complexity of adolescent substance use behaviors. Results may have limited applicability for broader prevention program development for Cherokee adolescents, as the remaining intervention targets were not strongly indicated. Findings contribute to the growing body of evidence needed to address substance misuse in rural and AI youth.

## Supplementary Material

Supp 1

Supp 2

Supplemental data for this article can be accessed online at https://doi.org/10.1080/29973368.2026.2667173.

## Figures and Tables

**Table 1.** Sample characteristics (n = 360).

Variable	n	%

Predictors		
I have seen it mess up others. I don’t want that for my life. (Mess up others)	205	56.94
I’m focused on the future. (Future focus)	188	53.56
I want to be healthy. (Healthy)	181	50.28
I have positive people in my life. (Positive people)	152	42.22
I do not want to get in trouble. (Trouble)	135	37.50
It gets in the way of things I want to do. (Gets in the way)	105	29.17
It makes me feel bad. (Feel bad)	47	13.06
**Covariates**		
Age		
15	181	54.03
16	142	42.39
>16	12	3.58
Gender		
Female	169	50.00
Male	158	46.75
Decline to answer	11	3.25
Race/ethnicity		
American Indian only	92	25.56
White only	138	38.33
American Indian and other	83	23.06
Other race/ethnicity	47	13.06
Free/reduced-price lunch	227	67.56
**Continuous covariates and outcomes**		
Variable	M	SD
Baseline		
Past 30-day alcohol use	0.26	1.37
Past 30-day cannabis use	0.42	2.93
Past 30-day prescription opioid misuse	0.15	0.98
Past 30-day vaping nicotine	1.15	4.65
Wave 3 (initial follow-up)		
Past 30-day alcohol use	0.90	4.06
Past 30-day cannabis use	1.56	5.99
Past 30-day prescription opioid misuse	0.35	3.03
Past 30-day vaping nicotine	1.68	6.04
Wave 4		
Past 30-day alcohol use	0.67	2.92
Past 30-day cannabis use	1.38	5.53
Past 30-day prescription opioid misuse	0.26	2.54
Past 30-day vaping nicotine	1.49	5.63
Wave 5		
Past 30-day alcohol use	0.71	2.98
Past 30-day cannabis use	0.68	3.82
Past 30-day prescription opioid misuse	0.14	1.82
Past 30-day vaping nicotine	1.10	4.98

*Notes*. Covariates come from wave 1 survey (Fall 2021). Motivations for nonuse come from Spring 2022 computerized Screening and Brief Intervention. Outcomes come from wave 3–5 surveys (Fall 2022, Spring 2023, and Fall 2023). Percentages were calculated on available data for each measure. All variables shown were used in multiple imputations.

**Table 2.** Longitudinal growth curve interaction effects (n = 360).

	Past 30-day alcohol use	Past 30-day cannabis use	Past 30-day rx opioid misuse	Past 30-day vaping nicotine
	Est. (SE)	Est. (SE)	Est. (SE)	Est. (SE)
Mess up others*wave	0.075 (0.277)	0.038 (0.245)	0.596 (0.573)	0.400 (0.236)
Future focus*wave	−0.207 (0.286)	0.196 (0.260)	0.266 (0.586)	0.142 (0.245)
Healthy*wave	−0.177 (0.313)	−0.025 (0.243)	0.190 (0.717)	0.321 (0.248)
Positive people*wave	−0.277 (0.278)	0.174 (0.299)	1.014 (0.626)	**0.796 (0.276)**
Trouble*wave	0.062 (0.283)	−0.081 (0.239)	0.421 (0.579)	0.107 (0.253)
Gets in the way*wave	0.099 (0.268)	0.057 (0.302)	1.162 (0.625)	0.473 (0.271)
Feel bad*wave	−0.075 (0.372)	−0.195 (0.413)	0.979 (0.600)	−0.150 (0.348)

*Notes.* Models adjusted for motivations for nonuse, age, race/ethnicity, gender, free or reduced-price lunch, and baseline use of the outcome substance; random intercept for school; compound-symmetry residual covariance structure to account for within-student repeated measures. Bold indicates statistical significance (p<.05).

**Table 3.** Main effects on initial follow-up (wave 3) outcomes (n = 360).

	Past 30-day alcohol use	Past 30-day cannabis use	Past 30-day rx opioid misuse	Past 30-day vaping nicotine
	Est. (SE)	Est. (SE)	Est. (SE)	Est. (SE)
Mess up others	−0.454 (0.538)	0.402 (0.467)	−0.611 (1.220)	−0.004 (0.385)
Future focus	0.421 (0.539)	−0.623 (0.502)	0.524 (1.187)	0.144 (0.410)
Healthy	0.993 (0.566)	0.144 (0.454)	1.484 (1.249)	0.310 (0.360)
Positive people	0.101 (0.551)	**−1.847 (0.670)**	−2.062 (1.842)	**−1.409 (0.530)**
Trouble	0.788 (0.521)	0.542 (0.425)	1.796 (1.256)	−0.295 (0.474)
Gets in the way	−0.056 (0.551)	−0.604 (0.589)	−0.066 (1.565)	0.773 (0.587)
Feel bad	−0.514 (0.758)	**1.226 (0.596)**	0.736 (1.697)	−0.074 (0.310)

*Notes.* Models adjusted for motivations for nonuse, age, race/ethnicity, gender, free or reduced-price lunch, and baseline use of the outcome substance (except for rx opioid misuse due to near-zero variance); random intercept for school. Bold indicates statistical significance (p < 0.05).
