# Supplementary material for: Motivations for Nonuse as Predictors of Substance Use Among Reservation-Based Youth in a Computerized Screening and Brief Intervention: The Power of Positive People
Source: J Child Adolesc Subst Use. Author manuscript; Available in PMC 2026 May 26. (PMC13200286; doi:10.1080/29973368.2026.2667173)
Supplement: Supp 1 [file NIHMS2175222-supplement-Supp_1.pdf]

Supplemental Table S0. Example: Mixed-effects model predicting past-30-day alcohol use from “I’ve seen it mess up others” motivation (waves 3–5)

Fixed Effects	Estimate	SE	95% CI	t	p
Intercept	−9.84	2.18	−14.12, −5.56	−4.51	<.001
Mess up others	−0.38	1.13	−2.61, 1.84	−0.34	0.734
Wave (continuous)	−0.35	0.22	−0.78, 0.08	−1.59	0.113
Mess up others x wave	0.08	0.28	−0.47, 0.62	0.27	0.786
Covariates					
Age	0.60	0.14	0.33, 0.87	4.36	<.001
Gender (male)	0.52	0.30	−0.08, 1.12	1.72	0.087
Race: AI only	−0.17	0.37	−0.90, 0.57	−0.45	0.652
Race: AI and other	0.23	0.33	−0.43, 0.88	0.68	0.499
Race: Other	−0.07	0.60	−1.25, 1.11	−0.12	0.905
Free/reduced lunch	−0.32	0.30	−0.91, 0.27	−1.08	0.281
Baseline alcohol use	0.04	0.06	−0.07, 0.16	0.74	0.461
Positive people	−0.27	0.34	−0.93, 0.40	−0.79	0.430
Future focused	−0.01	0.32	−0.63, 0.61	−0.03	0.972
Healthy	0.67	0.31	0.06, 1.28	2.15	0.032
Trouble	0.30	0.31	−0.30, 0.90	0.97	0.334
Gets in the way	0.29	0.35	−0.39, 0.97	0.84	0.403
Feel bad	−0.18	0.46	−1.08, 0.73	−0.39	0.700

**Random Effects/ Variance Components**	**Estimate**	**SE**
Student-level intercept variance	–	–
School-level intercept variance (σ^2)	0.44	0.34
Residual variance (CS structure)	1.19	0.35
Overdispersion (quasi-Poisson φ)	8.74	0.46

Note: Wave was coded 0, 1, and 2 corresponding to waves 3–5. A student-level random intercept was not included; within-student variability was modeled using a compound-symmetry residual structure. Fixed effects reflect pooled multiple-imputation estimates. Variance components (school-level intercept variance, residual variance, and overdispersion parameter) are drawn from one imputed dataset because SAS PROC MIANALYZE does not pool random-effects variances; these values were similar across imputations.
