# Supplementary material for: Motivations for Nonuse as Predictors of Substance Use Among Reservation-Based Youth in a Computerized Screening and Brief Intervention: The Power of Positive People
Source: J Child Adolesc Subst Use. Author manuscript; Available in PMC 2026 May 26. (PMC13200286; doi:10.1080/29973368.2026.2667173)
Supplement: Supp 2 [file NIHMS2175222-supplement-Supp_2.pdf]

Supplemental Tables S1–S28. Multiple-imputation–pooled fixed-effect estimates from Poisson mixed-effects models predicting monthly substance use

The MIANALYZE Procedure

Parameter Estimates (29 Imputations)							
Parameter	*Estimate*	*Std Error*	*95% Confidence Limits*		*DF*	*Minimum*	*Maximum*
INTERCEPT	-9.840007	2.181700	-14.1234	-5.55660	705.04	-11.562082	-7.794564
messupothers	-0.384571	1.133257	-2.6103	1.84117	586.04	-1.622967	0.593016
futurefoc	-0.011038	0.317552	-0.6342	0.61211	1001.2	-0.322061	0.241952
healthy	0.670442	0.311822	0.0582	1.28269	679.63	0.417585	0.963111
pospeo	-0.267424	0.338964	-0.9329	0.39806	717.26	-0.488591	0.223356
trouble	0.296480	0.306416	-0.3049	0.89789	857.54	0.016780	0.561906
getsintheway	0.290618	0.346882	-0.3906	0.97188	593.79	-0.109339	0.493864
feelbad	-0.176967	0.458697	-1.0801	0.72621	263.8	-0.855237	0.378319
age	0.599769	0.137660	0.3294	0.87017	556.08	0.453290	0.689566
gender_m	0.520478	0.302794	-0.0751	1.11606	340.96	0.297538	0.967659
race_cat_1	-0.169031	0.374208	-0.9049	0.56686	362.31	-0.542454	0.233361
race_cat_3	0.226430	0.334565	-0.4311	0.88392	452.14	-0.048546	0.632832
race_cat_4	-0.071160	0.597284	-1.2504	1.10809	165.98	-0.919981	0.729577
lunch_yes	-0.322866	0.298883	-0.9115	0.26580	248.43	-0.640511	0.067145
baseline_alc_mon	0.043637	0.059195	-0.0725	0.15979	1082.2	0.015113	0.105704
wave	-0.349340	0.220381	-0.7821	0.08343	629.51	-0.605005	-0.169800
*messupothers*wave*	0.075250	0.276751	-0.4687	0.61916	443.52	-0.138406	0.415036

Parameter Estimates (29 Imputations)			
Parameter	*Theta0*	*t for H0: Parameter=Theta0*	*Pr > |t|*
INTERCEPT	0	-4.51	<.0001
messupothers	0	-0.34	0.7345
futurefoc	0	-0.03	0.9723
healthy	0	2.15	0.0319
pospeo	0	-0.79	0.4304
trouble	0	0.97	0.3335
getsintheway	0	0.84	0.4025
feelbad	0	-0.39	0.7000
age	0	4.36	<.0001
gender_m	0	1.72	0.0865
race_cat_1	0	-0.45	0.6518
race_cat_3	0	0.68	0.4989
race_cat_4	0	-0.12	0.9053
lunch_yes	0	-1.08	0.2811
baseline_alc_mon	0	0.74	0.4612
wave	0	-1.59	0.1134
*messupothers*wave*	0	0.27	0.7858

The MIANALYZE Procedure

Parameter Estimates (29 Imputations)							
Parameter	*Estimate*	*Std Error*	*95% Confidence Limits*		*DF*	*Minimum*	*Maximum*
INTERCEPT	-10.304726	2.215687	-14.6571	-5.95237	543.75	-12.034752	-8.182164
messupothers	-0.081577	0.322567	-0.7146	0.55147	925.03	-0.359760	0.134540
futurefoc	0.820194	1.197163	-1.5347	3.17510	335.15	-1.006031	2.160128
healthy	0.676546	0.311886	0.0642	1.28889	702.22	0.422406	0.967410
pospeo	-0.262081	0.336174	-0.9220	0.39781	795.83	-0.489259	0.216494
trouble	0.291228	0.305312	-0.3079	0.89039	950.28	0.025415	0.547544
getsintheway	0.278179	0.345258	-0.3998	0.95615	641.03	-0.109957	0.478156
feelbad	-0.174686	0.457812	-1.0761	0.72669	267.29	-0.841149	0.391021
age	0.588633	0.139133	0.3153	0.86199	501.67	0.430010	0.678408
gender_m	0.512065	0.302785	-0.0835	1.10760	345.21	0.296261	0.953061
race_cat_1	-0.171890	0.375347	-0.9101	0.56629	355.46	-0.561520	0.222184
race_cat_3	0.224231	0.335486	-0.4351	0.88358	440.9	-0.043279	0.645613
race_cat_4	-0.109324	0.598425	-1.2904	1.07175	174.75	-0.939643	0.655332
lunch_yes	-0.309428	0.300389	-0.9011	0.28221	248	-0.636314	0.077634
baseline_alc_mon	0.043525	0.059437	-0.0731	0.16015	1087.5	0.015409	0.104589
wave	-0.187006	0.225736	-0.6309	0.25690	364.15	-0.463998	0.002552
*futurefoc*wave*	-0.207308	0.286039	-0.7699	0.35531	341.24	-0.481798	0.214600

Parameter Estimates (29 Imputations)			
Parameter	*Theta0*	*t for H0: Parameter=Theta0*	*Pr > |t|*
INTERCEPT	0	-4.65	<.0001
messupothers	0	-0.25	0.8004
futurefoc	0	0.69	0.4937
healthy	0	2.17	0.0304
pospeo	0	-0.78	0.4359
trouble	0	0.95	0.3404
getsintheway	0	0.81	0.4207
feelbad	0	-0.38	0.7031
age	0	4.23	<.0001
gender_m	0	1.69	0.0917
race_cat_1	0	-0.46	0.6473
race_cat_3	0	0.67	0.5042
race_cat_4	0	-0.18	0.8553
lunch_yes	0	-1.03	0.3040
baseline_alc_mon	0	0.73	0.4641
wave	0	-0.83	0.4080
*futurefoc*wave*	0	-0.72	0.4691

The MIANALYZE Procedure

Parameter Estimates (29 Imputations)							
Parameter	*Estimate*	*Std Error*	*95% Confidence Limits*		*DF*	*Minimum*	*Maximum*
INTERCEPT	-10.463845	2.244003	-14.8682	-6.05952	865.23	-12.431441	-8.490675
messupothers	-0.081660	0.324035	-0.7176	0.55428	907.09	-0.365571	0.137391
futurefoc	-0.009524	0.318322	-0.6342	0.61512	1009.1	-0.324629	0.243072
healthy	1.391471	1.288022	-1.1427	3.92561	317.28	0.032211	2.971213
pospeo	-0.271873	0.339555	-0.9385	0.39474	732.31	-0.492651	0.223097
trouble	0.294859	0.306729	-0.3071	0.89684	902.53	0.023241	0.560285
getsintheway	0.284862	0.345807	-0.3942	0.96390	647	-0.109486	0.481415
feelbad	-0.178865	0.459364	-1.0833	0.72559	265.61	-0.854523	0.379644
age	0.596475	0.136943	0.3275	0.86542	600.82	0.450669	0.687111
gender_m	0.528196	0.302555	-0.0668	1.12319	360.24	0.298391	0.950482
race_cat_1	-0.173109	0.376104	-0.9127	0.56653	359.69	-0.549732	0.221795
race_cat_3	0.224099	0.336931	-0.4381	0.88629	439.91	-0.046056	0.646421
race_cat_4	-0.084556	0.595146	-1.2591	1.09002	175.15	-0.925183	0.702111
lunch_yes	-0.312409	0.300877	-0.9050	0.28021	246.29	-0.638983	0.071571
baseline_alc_mon	0.044005	0.059134	-0.0720	0.16003	1111.5	0.015934	0.104947
wave	-0.183217	0.265119	-0.7047	0.33830	333.9	-0.465047	0.025157
*healthy*wave*	-0.177151	0.313364	-0.7942	0.43989	261.19	-0.582183	0.109131

Parameter Estimates (29 Imputations)			
Parameter	*Theta0*	*t for H0: Parameter=Theta0*	*Pr > |t|*
INTERCEPT	0	-4.66	<.0001
messupothers	0	-0.25	0.8011
futurefoc	0	-0.03	0.9761
healthy	0	1.08	0.2808
pospeo	0	-0.80	0.4236
trouble	0	0.96	0.3367
getsintheway	0	0.82	0.4104
feelbad	0	-0.39	0.6973
age	0	4.36	<.0001
gender_m	0	1.75	0.0817
race_cat_1	0	-0.46	0.6456
race_cat_3	0	0.67	0.5063
race_cat_4	0	-0.14	0.8872
lunch_yes	0	-1.04	0.3001
baseline_alc_mon	0	0.74	0.4569
wave	0	-0.69	0.4900
*healthy*wave*	0	-0.57	0.5723

The MIANALYZE Procedure

Parameter	Estimate	Std Error	95% Confidence Limits		DF	Minimum	Maximum
Parameter Estimates (29 Imputations)							
INTERCEPT	-10.360710	2.222476	-14.7262	-5.99527	558.04	-12.157437	-8.210795
messupothers	-0.094457	0.326327	-0.7349	0.54596	931.13	-0.362678	0.131216
futurefoc	-0.009467	0.319988	-0.6373	0.61841	1060.6	-0.321833	0.240354
healthy	0.672308	0.314758	0.0543	1.29027	714.99	0.415427	0.965047
pospeo	0.829228	1.117330	-1.3630	3.02143	1164.2	-0.110689	1.524760
trouble	0.299530	0.310591	-0.3101	0.90916	836.9	0.012719	0.568010
getsintheway	0.289390	0.349530	-0.3970	0.97579	621.44	-0.117049	0.499740
feelbad	-0.171879	0.461965	-1.0814	0.73765	268.84	-0.863195	0.376066
age	0.591027	0.139168	0.3177	0.86437	575.12	0.444996	0.674806
gender_m	0.520449	0.302161	-0.0736	1.11448	397.99	0.297988	0.919832
race_cat_1	-0.157887	0.376326	-0.8978	0.58200	389.26	-0.526944	0.240614
race_cat_3	0.228531	0.339714	-0.4391	0.89615	451.32	-0.047356	0.646224
race_cat_4	-0.102978	0.604600	-1.2962	1.09028	174.8	-0.944145	0.697303
lunch_yes	-0.307342	0.302257	-0.9026	0.28788	256.21	-0.628548	0.079098
baseline_alc_mon	0.044337	0.059800	-0.0730	0.16167	1123	0.015809	0.103949
wave	-0.183243	0.192653	-0.5613	0.19477	1097.1	-0.360271	-0.072628
pospeo*wave	-0.277120	0.277906	-0.8228	0.26858	651.09	-0.478341	0.015002

Parameter	Theta0	t for H0: Parameter=Theta0	Pr > |t|
Parameter Estimates (29 Imputations)			
INTERCEPT	0	-4.66	<.0001
messupothers	0	-0.29	0.7723
futurefoc	0	-0.03	0.9764
healthy	0	2.14	0.0330
pospeo	0	0.74	0.4581
trouble	0	0.96	0.3351
getsintheway	0	0.83	0.4080
feelbad	0	-0.37	0.7101
age	0	4.25	<.0001
gender_m	0	1.72	0.0858
race_cat_1	0	-0.42	0.6750
race_cat_3	0	0.67	0.5015
race_cat_4	0	-0.17	0.8650
lunch_yes	0	-1.02	0.3102
baseline_alc_mon	0	0.74	0.4586
wave	0	-0.95	0.3417
pospeo*wave	0	-1.00	0.3191

The MIANALYZE Procedure

Parameter Estimates (29 Imputations)

Parameter	*Estimate*	*Std Error*	*95% Confidence Limits*		*DF*	*Minimum*	*Maximum*
INTERCEPT	-9.845442	2.214959	-14.1953	-5.49555	608.92	-11.441390	-7.302050
messupothers	-0.081510	0.322005	-0.7135	0.55044	920.82	-0.360795	0.142515
futurefoc	-0.008232	0.316578	-0.6295	0.61299	1014.6	-0.310822	0.234068
healthy	0.671536	0.311536	0.0598	1.28324	673.41	0.413570	0.964550
pospeo	-0.268925	0.337845	-0.9322	0.39434	731.42	-0.489687	0.215788
trouble	0.045243	1.145494	-2.2069	2.29740	387.88	-1.351789	1.008377
getsintheway	0.291355	0.346873	-0.3899	0.97266	572.18	-0.106179	0.496760
feelbad	-0.176714	0.457582	-1.0777	0.72428	262.82	-0.839216	0.377840
age	0.597067	0.136956	0.3281	0.86606	572.05	0.448141	0.689981
gender_m	0.515603	0.301648	-0.0777	1.10888	349.16	0.292910	0.944330
race_cat_1	-0.167306	0.373486	-0.9018	0.56719	358.84	-0.543750	0.226513
race_cat_3	0.225585	0.334441	-0.4317	0.88289	439.47	-0.047207	0.638975
race_cat_4	-0.085979	0.596859	-1.2644	1.09242	166.24	-0.917338	0.714468
lunch_yes	-0.318123	0.300379	-0.9099	0.27366	234.35	-0.642018	0.077921
baseline_alc_mon	0.043559	0.059055	-0.0723	0.15944	1062.9	0.015057	0.104379
wave	-0.337013	0.202088	-0.7337	0.05968	785.39	-0.517128	-0.205680
*trouble*wave*	0.062468	0.282924	-0.4943	0.61919	306.38	-0.223018	0.356659

Parameter Estimates (29 Imputations)

Parameter	*Theta0*	*t for H0: Parameter=Theta0*	*Pr > |t|*
INTERCEPT	0	-4.44	<.0001
messupothers	0	-0.25	0.8002
futurefoc	0	-0.03	0.9793
healthy	0	2.16	0.0315
pospeo	0	-0.80	0.4263
trouble	0	0.04	0.9685
getsintheway	0	0.84	0.4013
feelbad	0	-0.39	0.6997
age	0	4.36	<.0001
gender_m	0	1.71	0.0883
race_cat_1	0	-0.45	0.6545
race_cat_3	0	0.67	0.5003
race_cat_4	0	-0.14	0.8856
lunch_yes	0	-1.06	0.2907
baseline_alc_mon	0	0.74	0.4609
wave	0	-1.67	0.0958
*trouble*wave*	0	0.22	0.8254

The MIANALYZE Procedure

Parameter Estimates (29 Imputations)							
Parameter	*Estimate*	*Std Error*	*95% Confidence Limits*		*DF*	*Minimum*	*Maximum*
INTERCEPT	-9.853113	2.155401	-14.0862	-5.62004	598.06	-11.359227	-7.835365
messupothers	-0.081650	0.321443	-0.7125	0.54919	926.09	-0.362954	0.137873
futurefoc	-0.006923	0.316119	-0.6272	0.61340	1009.5	-0.323638	0.240728
healthy	0.671424	0.310974	0.0608	1.28203	669.59	0.415828	0.965681
pospeo	-0.272746	0.336441	-0.9332	0.38772	756.13	-0.496070	0.212740
trouble	0.297779	0.304393	-0.2996	0.89519	890.19	0.029992	0.561557
getsintheway	-0.102055	1.115215	-2.2904	2.08630	1030.5	-1.456979	0.622784
feelbad	-0.176521	0.458179	-1.0788	0.72572	258.35	-0.853889	0.392702
age	0.598446	0.136981	0.3294	0.86750	562.7	0.458274	0.691610
gender_m	0.519809	0.301282	-0.0728	1.11241	341.23	0.294115	0.959902
race_cat_1	-0.167771	0.373263	-0.9019	0.56633	353.09	-0.542614	0.239662
race_cat_3	0.228273	0.334487	-0.4292	0.88573	425.27	-0.044174	0.642262
race_cat_4	-0.075627	0.592948	-1.2463	1.09507	165.87	-0.915594	0.719053
lunch_yes	-0.323882	0.300594	-0.9162	0.26840	228.81	-0.651863	0.079586
baseline_alc_mon	0.043149	0.059090	-0.0728	0.15910	1060.5	0.015097	0.104470
wave	-0.341406	0.182147	-0.6990	0.01618	738.16	-0.492485	-0.192259
*getsintheway*wave*	0.098803	0.268201	-0.4276	0.62519	880.8	-0.137950	0.390054

Parameter Estimates (29 Imputations)			
Parameter	*Theta0*	*t for H0: Parameter=Theta0*	*Pr > |t|*
INTERCEPT	0	-4.57	<.0001
messupothers	0	-0.25	0.7995
futurefoc	0	-0.02	0.9825
healthy	0	2.16	0.0312
pospeo	0	-0.81	0.4178
trouble	0	0.98	0.3282
getsintheway	0	-0.09	0.9271
feelbad	0	-0.39	0.7004
age	0	4.37	<.0001
gender_m	0	1.73	0.0854
race_cat_1	0	-0.45	0.6534
race_cat_3	0	0.68	0.4953
race_cat_4	0	-0.13	0.8987
lunch_yes	0	-1.08	0.2824
baseline_alc_mon	0	0.73	0.4654
wave	0	-1.87	0.0613
*getsintheway*wave*	0	0.37	0.7127

The MIANALYZE Procedure

Parameter Estimates (29 Imputations)							
Parameter	Estimate	Std Error	95% Confidence Limits		DF	Minimum	Maximum
INTERCEPT	-10.032750	2.173086	-14.3004	-5.76511	610.26	-11.632120	-8.020175
messupothers	-0.082205	0.323713	-0.7175	0.55308	939.48	-0.360223	0.136281
futurefoc	-0.013680	0.318189	-0.6380	0.61068	1050.9	-0.318139	0.237902
healthy	0.670162	0.312995	0.0556	1.28468	699.65	0.423859	0.962974
pospeo	-0.270305	0.338543	-0.9349	0.39427	774.75	-0.490038	0.216876
trouble	0.296795	0.306543	-0.3048	0.89840	915.43	0.024518	0.544909
getsintheway	0.290847	0.347899	-0.3924	0.97407	609.1	-0.114451	0.489440
feelbad	0.115695	1.475785	-2.7818	3.01323	695.01	-0.953429	1.841317
age	0.598214	0.137405	0.3284	0.86806	605.77	0.454804	0.690019
gender_m	0.517476	0.302545	-0.0775	1.11247	356.49	0.294838	0.957936
race_cat_1	-0.166209	0.375414	-0.9044	0.57203	366.21	-0.541840	0.233829
race_cat_3	0.228175	0.336881	-0.4339	0.89027	439.24	-0.051153	0.641715
race_cat_4	-0.073629	0.598859	-1.2559	1.10864	167.85	-0.937770	0.723577
lunch_yes	-0.319686	0.300468	-0.9115	0.27214	245.42	-0.640500	0.079002
baseline_alc_mon	0.043451	0.059485	-0.0733	0.16017	1099.5	0.014914	0.104462
wave	-0.293708	0.159550	-0.6068	0.01939	986.04	-0.395507	-0.157935
feelbad*wave	-0.074989	0.371582	-0.8054	0.65545	411.62	-0.547791	0.272012

Parameter Estimates (29 Imputations)			
Parameter	Theta0	t for H0: Parameter=Theta0	Pr > |t|
INTERCEPT	0	-4.62	<.0001
messupothers	0	-0.25	0.7996
futurefoc	0	-0.04	0.9657
healthy	0	2.14	0.0326
pospeo	0	-0.80	0.4249
trouble	0	0.97	0.3332
getsintheway	0	0.84	0.4035
feelbad	0	0.08	0.9375
age	0	4.35	<.0001
gender_m	0	1.71	0.0881
race_cat_1	0	-0.44	0.6582
race_cat_3	0	0.68	0.4986
race_cat_4	0	-0.12	0.9023
lunch_yes	0	-1.06	0.2884
baseline_alc_mon	0	0.73	0.4653
wave	0	-1.84	0.0659
feelbad*wave	0	-0.20	0.8402

The MIANALYZE Procedure

Parameter Estimates (27 Imputations)

Parameter	Estimate	Std Error	95% Confidence Limits		DF	Minimum	Maximum
INTERCEPT	-1.626522	2.783771	-7.10502	3.85198	295.9	-4.762126	1.413430
messupothers	0.099897	0.967908	-1.80202	2.00182	473.93	-0.631790	0.898214
futurefoc	-0.609103	0.404519	-1.40387	0.18567	498.99	-1.077125	-0.317993
healthy	0.133962	0.360849	-0.57446	0.84239	729.71	-0.352950	0.384283
pospeo	-1.040317	0.474655	-1.97323	-0.10741	433.83	-1.327490	-0.563693
trouble	0.498436	0.337754	-0.16445	1.16132	893.15	0.197394	0.794447
getsintheway	-0.419932	0.462001	-1.32618	0.48632	1473.5	-0.690054	0.044370
feelbad	0.519104	0.510353	-0.48167	1.51988	2427	0.199226	0.904049
age	0.179468	0.178664	-0.17216	0.53109	293.82	-0.072263	0.351798
gender_m	0.599047	0.355362	-0.09969	1.29778	377.81	0.207468	0.873015
race_cat_1	-0.576806	0.572868	-1.71256	0.55895	106.08	-1.441202	0.119088
race_cat_3	0.294578	0.343925	-0.38234	0.97150	288.19	-0.107765	0.597385
race_cat_4	0.310367	0.452486	-0.57889	1.19962	449.14	-0.098155	0.755737
lunch_yes	-0.181845	0.266584	-0.70527	0.34158	679.34	-0.414236	0.099071
baseline_thc_mon	0.091336	0.023144	0.04591	0.13676	905.25	0.070084	0.109765
wave	-0.350585	0.198432	-0.74037	0.03920	546.31	-0.498413	-0.137472
messupothers*wave	0.038416	0.245310	-0.44378	0.52061	417.32	-0.222058	0.237788

Parameter Estimates (27 Imputations)

Parameter	Theta0	t for H0: Parameter=Theta0	Pr > |t|
INTERCEPT	0	-0.58	0.5595
messupothers	0	0.10	0.9178
futurefoc	0	-1.51	0.1328
healthy	0	0.37	0.7106
pospeo	0	-2.19	0.0289
trouble	0	1.48	0.1404
getsintheway	0	-0.91	0.3635
feelbad	0	1.02	0.3092
age	0	1.00	0.3160
gender_m	0	1.69	0.0927
race_cat_1	0	-1.01	0.3163
race_cat_3	0	0.86	0.3924
race_cat_4	0	0.69	0.4931
lunch_yes	0	-0.68	0.4954
baseline_thc_mon	0	3.95	<.0001
wave	0	-1.77	0.0778
messupothers*wave	0	0.16	0.8756

The MIANALYZE Procedure

Parameter Estimates (27 Imputations)

Parameter	*Estimate*	*Std Error*	*95% Confidence*	*Limits*	*DF*	*Minimum*	*Maximum*
INTERCEPT	-1.701058	2.695852	-7.00501	3.60290	318.09	-4.502832	1.715871
messupothers	0.235900	0.364560	-0.48002	0.95182	622.09	-0.124416	0.487293
futurefoc	-1.328159	1.054558	-3.39965	0.74333	545.41	-2.367016	-0.422460
healthy	0.109803	0.362743	-0.60244	0.82204	675.24	-0.370660	0.395749
pospeo	-1.051830	0.476373	-1.98815	-0.11551	427.96	-1.327757	-0.573740
trouble	0.516167	0.337139	-0.14550	1.17783	902.23	0.250014	0.820147
getsintheway	-0.421904	0.466229	-1.33657	0.49276	1268.4	-0.709932	0.033511
feelbad	0.505074	0.519041	-0.51295	1.52310	1713	0.174758	0.908831
age	0.194404	0.180290	-0.16047	0.54928	283.38	-0.059920	0.359033
gender_m	0.621333	0.361286	-0.08941	1.33208	325.81	0.215090	0.904993
race_cat_1	-0.590933	0.581090	-1.74328	0.56142	103.81	-1.472725	0.125054
race_cat_3	0.290473	0.345586	-0.38984	0.97079	276.67	-0.119679	0.594089
race_cat_4	0.319627	0.448189	-0.56096	1.20021	495.71	-0.161058	0.730081
lunch_yes	-0.182544	0.265687	-0.70417	0.33908	712.22	-0.417189	0.113834
baseline_thc_mon	0.091928	0.023282	0.04623	0.13763	821.42	0.070606	0.111250
wave	-0.400487	0.175846	-0.74577	-0.05521	666.58	-0.523187	-0.197895
*futurefoc*wave*	0.196061	0.260178	-0.31503	0.70715	536.07	-0.037507	0.377687

Parameter Estimates (27 Imputations)

Parameter	*Theta0*	*t for H0: Parameter=Theta0*	*Pr > |t|*
INTERCEPT	0	-0.63	0.5285
messupothers	0	0.65	0.5178
futurefoc	0	-1.26	0.2084
healthy	0	0.30	0.7622
pospeo	0	-2.21	0.0278
trouble	0	1.53	0.1261
getsintheway	0	-0.90	0.3657
feelbad	0	0.97	0.3306
age	0	1.08	0.2818
gender_m	0	1.72	0.0864
race_cat_1	0	-1.02	0.3115
race_cat_3	0	0.84	0.4013
race_cat_4	0	0.71	0.4761
lunch_yes	0	-0.69	0.4923
baseline_thc_mon	0	3.95	<.0001
wave	0	-2.28	0.0231
*futurefoc*wave*	0	0.75	0.4514

The MIANALYZE Procedure

Parameter Estimates (27 Imputations)							
Parameter	Estimate	Std Error	95% Confidence Limits		DF	Minimum	Maximum
INTERCEPT	-1.787966	2.682957	-7.06432	3.48838	357.74	-4.311954	1.212583
messupothers	0.242856	0.365042	-0.47396	0.95967	644.1	-0.111037	0.502125
futurefoc	-0.601258	0.402383	-1.39167	0.18916	544.41	-1.074439	-0.324192
healthy	0.223797	0.944282	-1.63010	2.07769	715.94	-0.414893	1.130524
pospeo	-1.047429	0.477190	-1.98537	-0.10949	426.58	-1.328215	-0.572230
trouble	0.502138	0.337512	-0.16022	1.16450	946.16	0.218160	0.791803
getsintheway	-0.424399	0.463457	-1.33351	0.48471	1455.3	-0.687474	0.030106
feelbad	0.526818	0.509848	-0.47296	1.52659	2473.6	0.199589	0.918370
age	0.182519	0.177313	-0.16637	0.53141	309.81	-0.070302	0.340066
gender_m	0.601226	0.353695	-0.09409	1.29654	403.24	0.208861	0.874094
race_cat_1	-0.581880	0.576151	-1.72436	0.56060	104.38	-1.470129	0.112018
race_cat_3	0.293611	0.345499	-0.38653	0.97375	276.95	-0.116471	0.594817
race_cat_4	0.297626	0.452687	-0.59202	1.18727	449.24	-0.113201	0.747772
lunch_yes	-0.178092	0.268383	-0.70513	0.34895	625.62	-0.415743	0.100627
baseline_thc_mon	0.090860	0.023313	0.04510	0.13662	845.02	0.070207	0.109523
wave	-0.321394	0.188760	-0.69239	0.04960	434.96	-0.479847	-0.097178
healthy*wave	-0.024576	0.242559	-0.50106	0.45191	536.12	-0.251464	0.155870

Parameter Estimates (27 Imputations)			
Parameter	Theta0	t for H0: Parameter=Theta0	Pr > |t|
INTERCEPT	0	-0.67	0.5056
messupothers	0	0.67	0.5061
futurefoc	0	-1.49	0.1357
healthy	0	0.24	0.8127
pospeo	0	-2.19	0.0287
trouble	0	1.49	0.1371
getsintheway	0	-0.92	0.3600
feelbad	0	1.03	0.3016
age	0	1.03	0.3041
gender_m	0	1.70	0.0899
race_cat_1	0	-1.01	0.3149
race_cat_3	0	0.85	0.3962
race_cat_4	0	0.66	0.5112
lunch_yes	0	-0.66	0.5072
baseline_thc_mon	0	3.90	0.0001
wave	0	-1.70	0.0893
healthy*wave	0	-0.10	0.9193

The MIANALYZE Procedure

Parameter Estimates (27 Imputations)							
Parameter	Estimate	Std Error	95% Confidence	Limits	DF	Minimum	Maximum
INTERCEPT	-1.634703	2.673599	-6.89379	3.62438	336.29	-4.279972	1.566019
messupothers	0.243026	0.362380	-0.46846	0.95451	696.41	-0.114336	0.472019
futurefoc	-0.616732	0.402304	-1.40697	0.17351	549.75	-1.055648	-0.320555
healthy	0.143978	0.361369	-0.56551	0.85346	708.05	-0.344365	0.402581
pospeo	-1.690265	1.212192	-4.07177	0.69124	509.63	-2.985788	-0.697124
trouble	0.492253	0.334862	-0.16482	1.14933	1050.9	0.228176	0.764192
getsintheway	-0.425670	0.464460	-1.33681	0.48547	1355	-0.686695	0.045582
feelbad	0.523412	0.510069	-0.47680	1.52362	2457.7	0.190660	0.921861
age	0.184132	0.177037	-0.16416	0.53242	323.77	-0.069215	0.345341
gender_m	0.598872	0.353562	-0.09622	1.29396	396.19	0.213997	0.875984
race_cat_1	-0.578353	0.578907	-1.72649	0.56978	102.93	-1.468221	0.114750
race_cat_3	0.290133	0.346992	-0.39306	0.97332	266.97	-0.144051	0.597318
race_cat_4	0.324942	0.445948	-0.55104	1.20092	546.48	-0.100740	0.722757
lunch_yes	-0.180163	0.266180	-0.70276	0.34244	706.63	-0.410380	0.101287
baseline_thc_mon	0.091586	0.023118	0.04622	0.13696	934.91	0.070480	0.109414
wave	-0.371062	0.170374	-0.70587	-0.03625	458.38	-0.522244	-0.191547
pospeo*wave	0.174163	0.298645	-0.41281	0.76113	434.21	-0.085561	0.538490

Parameter Estimates (27 Imputations)			
Parameter	Theta0	t for H0: Parameter=Theta0	Pr > |t|
INTERCEPT	0	-0.61	0.5413
messupothers	0	0.67	0.5027
futurefoc	0	-1.53	0.1259
healthy	0	0.40	0.6904
pospeo	0	-1.39	0.1638
trouble	0	1.47	0.1419
getsintheway	0	-0.92	0.3596
feelbad	0	1.03	0.3049
age	0	1.04	0.2991
gender_m	0	1.69	0.0911
race_cat_1	0	-1.00	0.3201
race_cat_3	0	0.84	0.4038
race_cat_4	0	0.73	0.4665
lunch_yes	0	-0.68	0.4987
baseline_thc_mon	0	3.96	<.0001
wave	0	-2.18	0.0299
pospeo*wave	0	0.58	0.5601

The MIANALYZE Procedure

Parameter Estimates (27 Imputations)

Parameter	Estimate	Std Error	95% Confidence	Limits	DF	Minimum	Maximum
INTERCEPT	-1.879999	2.743788	-7.27783	3.51783	324.99	-4.502631	1.487031
messupothers	0.252276	0.364854	-0.46420	0.96876	629.09	-0.106506	0.496856
futurefoc	-0.611107	0.402105	-1.40097	0.17875	546.16	-1.063815	-0.324823
healthy	0.138179	0.362602	-0.57377	0.85013	682.64	-0.353642	0.394925
pospeo	-1.042339	0.471279	-1.96840	-0.11628	472.61	-1.317097	-0.575549
trouble	0.799464	0.923741	-1.01344	2.61237	910.74	0.000220	1.340845
getsintheway	-0.420063	0.460482	-1.32329	0.48317	1554.1	-0.693934	0.037728
feelbad	0.527157	0.508804	-0.47059	1.52490	2374.8	0.184421	0.910363
age	0.180416	0.178373	-0.17058	0.53141	305.6	-0.072016	0.338183
gender_m	0.595942	0.354283	-0.10062	1.29250	387.14	0.207137	0.860599
race_cat_1	-0.569510	0.573809	-1.70715	0.56813	105.92	-1.389848	0.126338
race_cat_3	0.311242	0.351276	-0.38053	1.00301	255.11	-0.131804	0.633351
race_cat_4	0.336347	0.458899	-0.56571	1.23841	414.26	-0.130076	0.828282
lunch_yes	-0.171444	0.269042	-0.69981	0.35692	605.62	-0.413315	0.115830
baseline_thc_mon	0.091344	0.023287	0.04564	0.13705	840.52	0.070519	0.109724
wave	-0.292732	0.191423	-0.66883	0.08336	498.13	-0.492015	-0.155841
trouble*wave	-0.080670	0.239475	-0.55093	0.38959	630.95	-0.278898	0.111954

Parameter Estimates (27 Imputations)

Parameter	Theta0	t for H0: Parameter=Theta0	Pr > |t|
INTERCEPT	0	-0.69	0.4937
messupothers	0	0.69	0.4895
futurefoc	0	-1.52	0.1291
healthy	0	0.38	0.7033
pospeo	0	-2.21	0.0275
trouble	0	0.87	0.3870
getsintheway	0	-0.91	0.3618
feelbad	0	1.04	0.3003
age	0	1.01	0.3126
gender_m	0	1.68	0.0934
race_cat_1	0	-0.99	0.3232
race_cat_3	0	0.89	0.3764
race_cat_4	0	0.73	0.4640
lunch_yes	0	-0.64	0.5242
baseline_thc_mon	0	3.92	<.0001
wave	0	-1.53	0.1268
trouble*wave	0	-0.34	0.7363

The MIANALYZE Procedure

Parameter Estimates (27 Imputations)

Parameter	Estimate	Std Error	95% Confidence Limits		DF	Minimum	Maximum
INTERCEPT	-1.687980	2.684868	-6.96987	3.59391	325.4	-4.606385	1.488515
messupothers	0.242151	0.364563	-0.47373	0.95803	643.55	-0.116122	0.485628
futurefoc	-0.606740	0.401564	-1.39549	0.18201	562.76	-1.056405	-0.316955
healthy	0.138126	0.361195	-0.57098	0.84723	732.05	-0.343155	0.405365
pospeo	-1.045961	0.474104	-1.97768	-0.11425	453.39	-1.322057	-0.573366
trouble	0.498555	0.336782	-0.16236	1.15947	961.76	0.232405	0.793371
getsintheway	-0.628943	1.181509	-2.94586	1.68798	2325.2	-1.537272	0.233007
feelbad	0.523668	0.511518	-0.47942	1.52676	2281.9	0.191490	0.907465
age	0.181014	0.178649	-0.17061	0.53264	287.54	-0.074199	0.361967
gender_m	0.593607	0.354534	-0.10346	1.29067	385.64	0.206519	0.875417
race_cat_1	-0.581094	0.578282	-1.72794	0.56575	103.28	-1.474278	0.110061
race_cat_3	0.295103	0.345626	-0.38528	0.97548	277.6	-0.119421	0.596083
race_cat_4	0.309698	0.448347	-0.57126	1.19065	481.97	-0.089547	0.746272
lunch_yes	-0.177362	0.268156	-0.70394	0.34922	634.25	-0.429228	0.109086
baseline_thc_mon	0.090891	0.023225	0.04531	0.13647	895.78	0.070091	0.109272
wave	-0.341610	0.165206	-0.66632	-0.01690	431.76	-0.482879	-0.149361
getsintheway*wave	0.056530	0.302093	-0.53634	0.64940	917.3	-0.220508	0.378757

Parameter Estimates (27 Imputations)

Parameter	Theta0	t for H0: Parameter=Theta0	Pr > |t|
INTERCEPT	0	-0.63	0.5300
messupothers	0	0.66	0.5068
futurefoc	0	-1.51	0.1314
healthy	0	0.38	0.7023
pospeo	0	-2.21	0.0279
trouble	0	1.48	0.1391
getsintheway	0	-0.53	0.5946
feelbad	0	1.02	0.3061
age	0	1.01	0.3118
gender_m	0	1.67	0.0949
race_cat_1	0	-1.00	0.3173
race_cat_3	0	0.85	0.3939
race_cat_4	0	0.69	0.4901
lunch_yes	0	-0.66	0.5086
baseline_thc_mon	0	3.91	<.0001
wave	0	-2.07	0.0393
getsintheway*wave	0	0.19	0.8516

The MIANALYZE Procedure

Parameter Estimates (27 Imputations)							
Parameter	Estimate	Std Error	95% Confidence Limits		DF	Minimum	Maximum
INTERCEPT	-2.109304	2.806471	-7.63448	3.41587	271.94	-5.130599	1.404116
messupothers	0.241541	0.363971	-0.47317	0.95625	646.82	-0.110221	0.496008
futurefoc	-0.601519	0.399558	-1.38627	0.18323	582.33	-1.060341	-0.328355
healthy	0.124864	0.361973	-0.58580	0.83553	710.16	-0.352031	0.399682
pospeo	-1.045202	0.473162	-1.97507	-0.11533	451.67	-1.333116	-0.575493
trouble	0.498703	0.335534	-0.15972	1.15713	1009.5	0.231740	0.781572
getsintheway	-0.430167	0.460218	-1.33285	0.47252	1613.6	-0.683856	0.025843
feelbad	1.242901	1.544420	-1.80103	4.28683	217.71	-0.680860	3.076817
age	0.201176	0.183538	-0.16019	0.56254	266.74	-0.071185	0.372598
gender_m	0.591783	0.355542	-0.10730	1.29087	378.58	0.176737	0.867002
race_cat_1	-0.568280	0.573947	-1.70626	0.56970	105.37	-1.434441	0.135070
race_cat_3	0.305419	0.346814	-0.37737	0.98821	271.08	-0.107342	0.609723
race_cat_4	0.325837	0.452491	-0.56354	1.21521	429.55	-0.089134	0.794392
lunch_yes	-0.185660	0.269258	-0.71445	0.34313	609.03	-0.411197	0.104795
baseline_thc_mon	0.091051	0.023309	0.04530	0.13680	839.13	0.070262	0.109635
wave	-0.318923	0.157325	-0.62803	-0.00982	498.81	-0.436482	-0.139445
feelbad*wave	-0.195359	0.412962	-1.01032	0.61961	176.97	-0.694909	0.354496

Parameter Estimates (27 Imputations)			
Parameter	Theta0	t for H0: Parameter=Theta0	Pr > |t|
INTERCEPT	0	-0.75	0.4530
messupothers	0	0.66	0.5072
futurefoc	0	-1.51	0.1327
healthy	0	0.34	0.7302
pospeo	0	-2.21	0.0277
trouble	0	1.49	0.1375
getsintheway	0	-0.93	0.3501
feelbad	0	0.80	0.4218
age	0	1.10	0.2740
gender_m	0	1.66	0.0968
race_cat_1	0	-0.99	0.3244
race_cat_3	0	0.88	0.3793
race_cat_4	0	0.72	0.4719
lunch_yes	0	-0.69	0.4908
baseline_thc_mon	0	3.91	0.0001
wave	0	-2.03	0.0432
feelbad*wave	0	-0.47	0.6367

The MIANALYZE Procedure

Parameter Estimates (30 Imputations)

Parameter	*Estimate*	*Std Error*	*95% Confidence Limits*		*DF*	*Minimum*	*Maximum*
INTERCEPT	-6.612020	3.033994	-12.5656	-0.65839	1013.4	-10.418701	-5.107294
messupothers	-2.179478	2.153886	-6.4037	2.04470	1939.3	-4.456925	-0.878496
futurefoc	-0.299351	0.556123	-1.3903	0.79164	1302.7	-0.601776	0.317891
healthy	1.107324	0.556962	0.0137	2.20095	660.9	0.311096	1.645421
pospeo	-0.570976	0.622503	-1.7933	0.65132	668.67	-1.527947	-0.021127
trouble	0.701822	0.525602	-0.3297	1.73339	886.66	-0.014281	1.051998
getsintheway	0.701668	0.528745	-0.3351	1.73846	2660.9	0.290553	1.021649
feelbad	-0.038428	0.743262	-1.5011	1.42426	298.9	-0.470767	1.117883
age	0.421778	0.190829	0.0471	0.79646	680.75	0.178287	0.659571
gender_m	0.093295	0.443649	-0.7766	0.96318	3067	-0.197573	0.432139
race_cat_1	-1.844759	0.981872	-3.7778	0.08833	270.26	-2.646752	-0.350498
race_cat_3	-0.182904	0.523543	-1.2102	0.84435	1101.9	-0.554822	0.239619
race_cat_4	-1.499708	1.402681	-4.2666	1.26722	188.97	-2.347698	0.820413
lunch_yes	-0.022187	0.527757	-1.0606	1.01620	313.78	-0.545878	0.381410
baseline_rx_op_mon	0.313302	0.144365	0.0295	0.59706	422.98	0.039380	0.404937
wave	-0.868294	0.490196	-1.8306	0.09397	778.71	-1.144879	-0.316835
*messupothers*wave*	0.595641	0.572757	-0.5282	1.71948	1083.6	0.243498	1.236892

Parameter Estimates (30 Imputations)

Parameter	*Theta0*	*t for H0: Parameter=Theta0*	*Pr > |t|*
INTERCEPT	0	-2.18	0.0295
messupothers	0	-1.01	0.3117
futurefoc	0	-0.54	0.5905
healthy	0	1.99	0.0472
pospeo	0	-0.92	0.3594
trouble	0	1.34	0.1821
getsintheway	0	1.33	0.1846
feelbad	0	-0.05	0.9588
age	0	2.21	0.0274
gender_m	0	0.21	0.8335
race_cat_1	0	-1.88	0.0613
race_cat_3	0	-0.35	0.7269
race_cat_4	0	-1.07	0.2864
lunch_yes	0	-0.04	0.9665
baseline_rx_op_mon	0	2.17	0.0305
wave	0	-1.77	0.0769
*messupothers*wave*	0	1.04	0.2986

The MIANALYZE Procedure

Parameter Estimates (30 Imputations)

Parameter	Estimate	Std Error	95% Confidence Limits		DF	Minimum	Maximum
INTERCEPT	-7.193061	3.171124	-13.4206	-0.96557	617.58	-11.630779	-5.399969
messupothers	0.059419	0.579033	-1.0766	1.19547	1174.4	-0.354611	0.531841
futurefoc	-1.290285	2.225748	-5.6601	3.07951	713.82	-4.070153	1.025447
healthy	1.084215	0.561471	-0.0180	2.18646	750.16	0.313404	1.612699
pospeo	-0.572205	0.631232	-1.8115	0.66713	700.39	-1.551522	-0.023740
trouble	0.718017	0.530961	-0.3239	1.75995	995.16	0.019490	1.061929
getsintheway	0.704823	0.537934	-0.3500	1.75964	2646.4	0.281458	1.025460
feelbad	-0.040745	0.751986	-1.5204	1.43886	311.86	-0.480554	1.126655
age	0.407530	0.193144	0.0283	0.78678	661.64	0.162140	0.646576
gender_m	0.093472	0.447345	-0.7835	0.97049	4446.7	-0.183669	0.361396
race_cat_1	-1.848139	0.994024	-3.8049	0.10861	278.77	-2.630860	-0.326129
race_cat_3	-0.179409	0.531485	-1.2221	0.86333	1208.2	-0.581359	0.248610
race_cat_4	-1.487154	1.407848	-4.2629	1.28857	204.9	-2.423951	0.735155
lunch_yes	-0.012777	0.529914	-1.0550	1.02944	349.9	-0.521396	0.401167
baseline_rx_op_mon	0.307399	0.145555	0.0214	0.59344	455.94	0.038893	0.398090
wave	-0.640392	0.469191	-1.5630	0.28222	369.67	-0.989006	0.024380
futurefoc*wave	0.265983	0.586354	-0.8862	1.41817	472.63	-0.389045	1.072699

Parameter Estimates (30 Imputations)

Parameter	Theta0	t for H0: Parameter=Theta0	Pr > |t|
INTERCEPT	0	-2.27	0.0237
messupothers	0	0.10	0.9183
futurefoc	0	-0.58	0.5623
healthy	0	1.93	0.0539
pospeo	0	-0.91	0.3650
trouble	0	1.35	0.1766
getsintheway	0	1.31	0.1902
feelbad	0	-0.05	0.9568
age	0	2.11	0.0352
gender_m	0	0.21	0.8345
race_cat_1	0	-1.86	0.0640
race_cat_3	0	-0.34	0.7358
race_cat_4	0	-1.06	0.2921
lunch_yes	0	-0.02	0.9808
baseline_rx_op_mon	0	2.11	0.0352
wave	0	-1.36	0.1731
futurefoc*wave	0	0.45	0.6503

The MIANALYZE Procedure

Parameter Estimates (30 Imputations)							
Parameter	Estimate	Std Error	95% Confidence Limits		DF	Minimum	Maximum
INTERCEPT	-7.069310	3.711089	-14.3618	0.223150	468.01	-12.974585	-4.981393
messupothers	0.059358	0.584763	-1.0879	1.206574	1261.2	-0.342800	0.521551
futurefoc	-0.295963	0.566826	-1.4077	0.815810	1653.9	-0.622095	0.253863
healthy	0.393783	2.646720	-4.8055	5.593102	531.34	-2.757156	3.950619
pospeo	-0.567678	0.637985	-1.8202	0.684885	709.66	-1.553989	-0.017735
trouble	0.719078	0.535561	-0.3318	1.769937	1079.4	0.022986	1.064512
getsintheway	0.706459	0.544780	-0.3618	1.774671	2793.2	0.273479	1.021042
feelbad	-0.031270	0.759753	-1.5261	1.463537	316.43	-0.457381	1.194860
age	0.399429	0.193233	0.0201	0.778790	727.32	0.174841	0.646642
gender_m	0.071975	0.454901	-0.8199	0.963860	3651.9	-0.220830	0.413481
race_cat_1	-1.840639	1.004866	-3.8184	0.137148	288.89	-2.620802	-0.262886
race_cat_3	-0.183770	0.539929	-1.2432	0.875611	1124.9	-0.588006	0.233397
race_cat_4	-1.477292	1.420914	-4.2784	1.323850	209.25	-2.384183	0.787411
lunch_yes	-0.009703	0.532342	-1.0564	1.037038	375.89	-0.505765	0.404424
baseline_rx_op_mon	0.305616	0.146439	0.0179	0.593362	476.02	0.037951	0.396584
wave	-0.635847	0.637666	-1.8898	0.618095	366.61	-1.012022	0.334829
healthy*wave	0.190188	0.717229	-1.2202	1.600545	369.97	-0.885245	1.118233

Parameter Estimates (30 Imputations)			
Parameter	Theta0	t for H0: Parameter=Theta0	Pr > |t|
INTERCEPT	0	-1.90	0.0574
messupothers	0	0.10	0.9192
futurefoc	0	-0.52	0.6016
healthy	0	0.15	0.8818
pospeo	0	-0.89	0.3739
trouble	0	1.34	0.1797
getsintheway	0	1.30	0.1948
feelbad	0	-0.04	0.9672
age	0	2.07	0.0391
gender_m	0	0.16	0.8743
race_cat_1	0	-1.83	0.0680
race_cat_3	0	-0.34	0.7336
race_cat_4	0	-1.04	0.2997
lunch_yes	0	-0.02	0.9855
baseline_rx_op_mon	0	2.09	0.0374
wave	0	-1.00	0.3194
healthy*wave	0	0.27	0.7910

The MIANALYZE Procedure

Parameter Estimates (30 Imputations)							
Parameter	Estimate	Std Error	95% Confidence	Limits	DF	Minimum	Maximum
INTERCEPT	-6.458354	3.142991	-12.6348	-0.28192	458.89	-10.710057	-4.669844
messupothers	0.081774	0.576592	-1.0496	1.21311	1104.7	-0.357163	0.545043
futurefoc	-0.294382	0.556301	-1.3857	0.79689	1402.9	-0.599833	0.267624
healthy	1.100386	0.560053	0.0006	2.20015	639.13	0.303180	1.621363
pospeo	-4.427360	2.353132	-9.0486	0.19390	607.75	-6.123857	-1.725654
trouble	0.696519	0.523999	-0.3317	1.72478	1001.9	0.035822	1.040744
getsintheway	0.703230	0.529450	-0.3349	1.74140	2708.2	0.304515	1.026317
feelbad	-0.052017	0.749751	-1.5278	1.42375	284.39	-0.497691	1.126780
age	0.441081	0.190386	0.0673	0.81483	758.35	0.178311	0.654391
gender_m	0.086426	0.444774	-0.7857	0.95852	2997.1	-0.191410	0.427691
race_cat_1	-1.851000	0.994760	-3.8100	0.10803	253.89	-2.603179	-0.274972
race_cat_3	-0.135522	0.525655	-1.1670	0.89596	1022.5	-0.554009	0.304142
race_cat_4	-1.503412	1.422999	-4.3113	1.30451	179.85	-2.468314	0.858587
lunch_yes	-0.049970	0.547127	-1.1278	1.02782	239.57	-0.560631	0.428954
baseline_rx_op_mon	0.312996	0.145919	0.0261	0.59988	389.67	0.036138	0.408993
wave	-1.010475	0.526734	-2.0510	0.03003	154.88	-1.460573	-0.233816
pospeo*wave	1.014132	0.625840	-0.2171	2.24539	321.23	0.179733	1.437799

Parameter Estimates (30 Imputations)			
Parameter	Theta0	t for H0: Parameter=Theta0	Pr > |t|
INTERCEPT	0	-2.05	0.0405
messupothers	0	0.14	0.8872
futurefoc	0	-0.53	0.5968
healthy	0	1.96	0.0499
pospeo	0	-1.88	0.0604
trouble	0	1.33	0.1841
getsintheway	0	1.33	0.1842
feelbad	0	-0.07	0.9447
age	0	2.32	0.0208
gender_m	0	0.19	0.8459
race_cat_1	0	-1.86	0.0639
race_cat_3	0	-0.26	0.7966
race_cat_4	0	-1.06	0.2922
lunch_yes	0	-0.09	0.9273
baseline_rx_op_mon	0	2.14	0.0326
wave	0	-1.92	0.0569
pospeo*wave	0	1.62	0.1061

The MIANALYZE Procedure

Parameter Estimates (30 Imputations)

Parameter	*Estimate*	*Std Error*	*95% Confidence*	*Limits*	*DF*	*Minimum*	*Maximum*
INTERCEPT	-6.716656	3.366982	-13.3315	-0.10176	508.96	-11.312855	-3.754693
messupothers	0.039697	0.582113	-1.1023	1.18168	1300	-0.340269	0.494507
futurefoc	-0.285124	0.565304	-1.3939	0.82363	1733.6	-0.603718	0.249070
healthy	1.075903	0.566734	-0.0366	2.18838	790.43	0.299360	1.647064
pospeo	-0.565113	0.635276	-1.8123	0.68207	732.42	-1.523430	-0.014574
trouble	-0.863219	2.180826	-5.1410	3.41453	1517.6	-3.340231	0.695940
getsintheway	0.715497	0.543565	-0.3504	1.78135	2639.7	0.284762	1.038648
feelbad	-0.030798	0.753935	-1.5141	1.45246	322.23	-0.468634	1.147387
age	0.403892	0.192325	0.0263	0.78150	696.03	0.175158	0.648999
gender_m	0.053981	0.450798	-0.8298	0.93775	4867	-0.222412	0.297808
race_cat_1	-1.848650	1.003478	-3.8239	0.12661	281.77	-2.636856	-0.303462
race_cat_3	-0.196246	0.532877	-1.2416	0.84913	1322.6	-0.559809	0.210800
race_cat_4	-1.510735	1.415198	-4.3003	1.27883	213.3	-2.409524	0.706495
lunch_yes	-0.021886	0.539428	-1.0830	1.03924	332.27	-0.568935	0.427262
baseline_rx_op_mon	0.307155	0.146159	0.0200	0.59436	472.83	0.042427	0.392351
wave	-0.740174	0.476412	-1.6759	0.19555	575.14	-0.985755	0.076992
*trouble*wave*	0.421285	0.579084	-0.7153	1.55787	857.08	-0.157069	1.137579

Parameter Estimates (30 Imputations)

Parameter	*Theta0*	*t for H0: Parameter=Theta0*	*Pr > |t|*
INTERCEPT	0	-1.99	0.0466
messupothers	0	0.07	0.9456
futurefoc	0	-0.50	0.6141
healthy	0	1.90	0.0580
pospeo	0	-0.89	0.3740
trouble	0	-0.40	0.6923
getsintheway	0	1.32	0.1882
feelbad	0	-0.04	0.9674
age	0	2.10	0.0361
gender_m	0	0.12	0.9047
race_cat_1	0	-1.84	0.0665
race_cat_3	0	-0.37	0.7127
race_cat_4	0	-1.07	0.2869
lunch_yes	0	-0.04	0.9677
baseline_rx_op_mon	0	2.10	0.0361
wave	0	-1.55	0.1208
*trouble*wave*	0	0.73	0.4671

The MIANALYZE Procedure

Parameter Estimates (30 Imputations)

Parameter	Estimate	Std Error	95% Confidence Limits		DF	Minimum	Maximum
INTERCEPT	-6.450908	3.056964	-12.4574	-0.44441	486.01	-10.406060	-4.797302
messupothers	0.080592	0.574196	-1.0463	1.20746	931.95	-0.377160	0.568727
futurefoc	-0.277933	0.549629	-1.3561	0.80025	1397.8	-0.590313	0.269504
healthy	1.102576	0.555902	0.0107	2.19449	558.06	0.289327	1.586571
pospeo	-0.582207	0.614402	-1.7885	0.62411	692.72	-1.527438	-0.033371
trouble	0.727047	0.519996	-0.2936	1.74765	861.34	0.017597	1.059658
getsintheway	-3.743584	2.392558	-8.4449	0.95771	475.3	-5.199602	-0.243927
feelbad	-0.048685	0.753226	-1.5317	1.43436	266.02	-0.473746	1.132797
age	0.447549	0.187682	0.0791	0.81601	730.68	0.178307	0.661907
gender_m	0.113053	0.440604	-0.7509	0.97697	2958.6	-0.178079	0.432430
race_cat_1	-1.879052	0.993553	-3.8366	0.07852	231.27	-2.696152	-0.278715
race_cat_3	-0.196287	0.523980	-1.2247	0.83209	889.26	-0.565926	0.271454
race_cat_4	-1.657044	1.434119	-4.4891	1.17497	161.72	-2.646847	0.795108
lunch_yes	-0.126488	0.560856	-1.2321	0.97917	209.14	-0.610017	0.384855
baseline_rx_op_mon	0.323206	0.147637	0.0328	0.61366	322.61	0.024741	0.428387
wave	-1.041507	0.507041	-2.0433	-0.03975	151.95	-1.495666	-0.234134
getsintheway*wave	1.162486	0.624803	-0.0669	2.39190	308.72	0.251377	1.620268

Parameter Estimates (30 Imputations)

Parameter	Theta0	t for H0: Parameter=Theta0	Pr > |t|
INTERCEPT	0	-2.11	0.0353
messupothers	0	0.14	0.8884
futurefoc	0	-0.51	0.6132
healthy	0	1.98	0.0478
pospeo	0	-0.95	0.3437
trouble	0	1.40	0.1624
getsintheway	0	-1.56	0.1183
feelbad	0	-0.06	0.9485
age	0	2.38	0.0174
gender_m	0	0.26	0.7975
race_cat_1	0	-1.89	0.0598
race_cat_3	0	-0.37	0.7080
race_cat_4	0	-1.16	0.2496
lunch_yes	0	-0.23	0.8218
baseline_rx_op_mon	0	2.19	0.0293
wave	0	-2.05	0.0417
getsintheway*wave	0	1.86	0.0638

The MIANALYZE Procedure

Parameter Estimates (30 Imputations)							
Parameter	Estimate	Std Error	95% Confidence	Limits	DF	Minimum	Maximum
INTERCEPT	-7.110421	2.864715	-12.7326	-1.48824	914.45	-10.063262	-5.646716
messupothers	0.092048	0.563324	-1.0134	1.19748	996.99	-0.329764	0.545564
futurefoc	-0.278515	0.545222	-1.3481	0.79108	1321.9	-0.584489	0.303175
healthy	1.089064	0.548294	0.0123	2.16585	606.45	0.288259	1.651779
pospeo	-0.573498	0.618476	-1.7882	0.64117	593.94	-1.565446	-0.003490
trouble	0.705154	0.516301	-0.3083	1.71856	831.57	0.007129	1.057019
getsintheway	0.726969	0.515377	-0.2836	1.73752	2838.9	0.347514	1.036381
feelbad	-3.909483	2.475987	-8.7660	0.94700	1614.4	-4.905755	-0.947646
age	0.427927	0.186348	0.0619	0.79393	578.45	0.175660	0.674741
gender_m	0.089140	0.435923	-0.7656	0.94389	2924.9	-0.210937	0.419708
race_cat_1	-1.863621	0.973296	-3.7804	0.05317	253.23	-2.633142	-0.385088
race_cat_3	-0.156765	0.509520	-1.1566	0.84306	1024.7	-0.540839	0.294079
race_cat_4	-1.574939	1.379926	-4.2983	1.14839	176	-2.503366	0.795569
lunch_yes	-0.059979	0.559268	-1.1631	1.04311	192.21	-0.592760	0.486562
baseline_rx_op_mon	0.317974	0.143775	0.0352	0.60071	363.01	0.031344	0.418143
wave	-0.769467	0.366540	-1.4905	-0.04848	337.58	-0.990855	-0.187717
feelbad*wave	0.979062	0.600000	-0.1984	2.15648	989.49	0.195552	1.341557

Parameter Estimates (30 Imputations)			
Parameter	Theta0	t for H0: Parameter=Theta0	Pr > |t|
INTERCEPT	0	-2.48	0.0132
messupothers	0	0.16	0.8702
futurefoc	0	-0.51	0.6096
healthy	0	1.99	0.0475
pospeo	0	-0.93	0.3542
trouble	0	1.37	0.1724
getsintheway	0	1.41	0.1585
feelbad	0	-1.58	0.1145
age	0	2.30	0.0220
gender_m	0	0.20	0.8380
race_cat_1	0	-1.91	0.0567
race_cat_3	0	-0.31	0.7584
race_cat_4	0	-1.14	0.2553
lunch_yes	0	-0.11	0.9147
baseline_rx_op_mon	0	2.21	0.0276
wave	0	-2.10	0.0365
feelbad*wave	0	1.63	0.1030

The MIANALYZE Procedure

Parameter Estimates (29 Imputations)							
Parameter	Estimate	Std Error	95% Confidence	Limits	DF	Minimum	Maximum
INTERCEPT	-4.187788	2.319327	-8.74299	0.36741	586.31	-5.519874	-0.672700
messupothers	-1.458503	0.937147	-3.29815	0.38114	775.53	-2.137065	-0.460600
futurefoc	-0.280584	0.323565	-0.91607	0.35490	586.57	-0.573479	0.049441
healthy	0.352078	0.285467	-0.20803	0.91219	1117.7	0.134897	0.627187
pospeo	-0.427450	0.337408	-1.09015	0.23525	575.24	-0.712456	-0.120797
trouble	-0.214978	0.305720	-0.81525	0.38529	677.74	-0.435919	0.179354
getsintheway	0.077707	0.351341	-0.61204	0.76746	734.69	-0.280809	0.377051
feelbad	0.191779	0.435393	-0.66373	1.04729	479.83	-0.424027	0.623612
age	0.375630	0.157658	0.06545	0.68581	318.9	0.108105	0.481005
gender_m	0.419098	0.289009	-0.14915	0.98734	382.36	0.097362	0.639492
race_cat_1	-0.106713	0.351891	-0.79992	0.58649	238.81	-0.531751	0.258745
race_cat_3	-0.007813	0.323253	-0.64364	0.62801	340.67	-0.311252	0.407868
race_cat_4	-0.023963	0.538563	-1.08785	1.03992	154.69	-0.734997	0.451355
lunch_yes	-0.175136	0.243510	-0.65331	0.30304	641.53	-0.398968	0.011999
baseline_vape_mon	0.079029	0.014547	0.05047	0.10759	765.15	0.069559	0.090744
wave	-0.528694	0.213961	-0.94971	-0.10768	307.42	-0.673357	-0.218586
messupothers*wave	0.400117	0.235890	-0.06321	0.86344	565.54	0.160162	0.579002

Parameter Estimates (29 Imputations)			
Parameter	Theta0	t for H0: Parameter=Theta0	Pr > |t|
INTERCEPT	0	-1.81	0.0715
messupothers	0	-1.56	0.1200
futurefoc	0	-0.87	0.3862
healthy	0	1.23	0.2177
pospeo	0	-1.27	0.2057
trouble	0	-0.70	0.4822
getsintheway	0	0.22	0.8250
feelbad	0	0.44	0.6598
age	0	2.38	0.0178
gender_m	0	1.45	0.1478
race_cat_1	0	-0.30	0.7620
race_cat_3	0	-0.02	0.9807
race_cat_4	0	-0.04	0.9646
lunch_yes	0	-0.72	0.4723
baseline_vape_mon	0	5.43	<.0001
wave	0	-2.47	0.0140
messupothers*wave	0	1.70	0.0904

The MIANALYZE Procedure

Parameter Estimates (29 Imputations)

Parameter	*Estimate*	*Std Error*	*95% Confidence Limits*		*DF*	*Minimum*	*Maximum*
INTERCEPT	-5.155013	2.325800	-9.72532	-0.58471	467.99	-6.592031	-1.585689
messupothers	0.121782	0.303623	-0.47406	0.71763	951.96	-0.118480	0.358386
futurefoc	-0.895188	0.992705	-2.84309	1.05271	1054.7	-1.675928	-0.393941
healthy	0.347348	0.290685	-0.22301	0.91771	1100.9	0.140118	0.645036
pospeo	-0.462444	0.342100	-1.13423	0.20935	631.29	-0.739878	-0.164608
trouble	-0.204126	0.308303	-0.80939	0.40114	728.67	-0.420878	0.186484
getsintheway	0.070448	0.355788	-0.62796	0.76885	788.2	-0.278786	0.358957
feelbad	0.221047	0.431909	-0.62724	1.06933	583.58	-0.385383	0.636320
age	0.395988	0.161225	0.07882	0.71316	326.86	0.132827	0.502014
gender_m	0.410125	0.290106	-0.15998	0.98023	458.66	0.085229	0.605873
race_cat_1	-0.128662	0.360909	-0.83942	0.58209	253.9	-0.527368	0.220192
race_cat_3	-0.017799	0.333089	-0.67301	0.63742	334.3	-0.339341	0.399286
race_cat_4	-0.003056	0.529932	-1.04859	1.04247	183.85	-0.648756	0.473744
lunch_yes	-0.186116	0.250374	-0.67774	0.30551	659.69	-0.394757	0.010010
baseline_vape_mon	0.079052	0.014580	0.05043	0.10767	811.21	0.069935	0.091992
wave	-0.357619	0.190961	-0.73301	0.01778	406.22	-0.516645	-0.087285
*futurefoc*wave*	0.141823	0.245373	-0.33993	0.62357	705.64	-0.021973	0.374606

Parameter Estimates (29 Imputations)

Parameter	*Theta0*	*t for H0: Parameter=Theta0*	*Pr > |t|*
INTERCEPT	0	-2.22	0.0271
messupothers	0	0.40	0.6884
futurefoc	0	-0.90	0.3674
healthy	0	1.19	0.2324
pospeo	0	-1.35	0.1769
trouble	0	-0.66	0.5081
getsintheway	0	0.20	0.8431
feelbad	0	0.51	0.6090
age	0	2.46	0.0146
gender_m	0	1.41	0.1581
race_cat_1	0	-0.36	0.7218
race_cat_3	0	-0.05	0.9574
race_cat_4	0	-0.01	0.9954
lunch_yes	0	-0.74	0.4575
baseline_vape_mon	0	5.42	<.0001
wave	0	-1.87	0.0618
*futurefoc*wave*	0	0.58	0.5635

The MIANALYZE Procedure

Parameter Estimates (29 Imputations)							
Parameter	Estimate	Std Error	95% Confidence	Limits	DF	Minimum	Maximum
INTERCEPT	-4.619327	2.382694	-9.30210	0.06345	443.41	-6.136232	-1.165009
messupothers	0.115522	0.304528	-0.48208	0.71312	979.75	-0.124345	0.343230
futurefoc	-0.329894	0.324138	-0.96609	0.30630	857.54	-0.606357	-0.023256
healthy	-0.904139	0.984638	-2.83762	1.02934	645.05	-1.716177	-0.131281
pospeo	-0.456652	0.344643	-1.13353	0.22023	588.78	-0.750650	-0.162846
trouble	-0.213163	0.307980	-0.81772	0.39139	791.91	-0.426074	0.163861
getsintheway	0.067496	0.356820	-0.63288	0.76787	829.07	-0.274892	0.367486
feelbad	0.242167	0.432050	-0.60637	1.09071	591.42	-0.376505	0.626270
age	0.392130	0.162499	0.07235	0.71191	301.67	0.118509	0.501605
gender_m	0.400564	0.291498	-0.17228	0.97340	457.76	0.090050	0.612567
race_cat_1	-0.101150	0.365089	-0.82038	0.61808	237.49	-0.491777	0.302770
race_cat_3	-0.009736	0.331965	-0.66251	0.64304	369.65	-0.326714	0.376212
race_cat_4	0.031736	0.529242	-1.01226	1.07573	188.56	-0.629772	0.482535
lunch_yes	-0.190768	0.250529	-0.68266	0.30112	692.3	-0.396919	-0.003058
baseline_vape_mon	0.079264	0.014498	0.05081	0.10772	877.4	0.070951	0.092520
wave	-0.481658	0.226433	-0.92760	-0.03572	252.62	-0.677438	-0.131334
healthy*wave	0.320911	0.247719	-0.16589	0.80772	458.68	0.124625	0.512436

Parameter Estimates (29 Imputations)			
Parameter	Theta0	t for H0: Parameter=Theta0	Pr > |t|
INTERCEPT	0	-1.94	0.0532
messupothers	0	0.38	0.7045
futurefoc	0	-1.02	0.3091
healthy	0	-0.92	0.3588
pospeo	0	-1.33	0.1857
trouble	0	-0.69	0.4891
getsintheway	0	0.19	0.8500
feelbad	0	0.56	0.5753
age	0	2.41	0.0164
gender_m	0	1.37	0.1701
race_cat_1	0	-0.28	0.7820
race_cat_3	0	-0.03	0.9766
race_cat_4	0	0.06	0.9522
lunch_yes	0	-0.76	0.4466
baseline_vape_mon	0	5.47	<.0001
wave	0	-2.13	0.0344
healthy*wave	0	1.30	0.1958

The MIANALYZE Procedure

Parameter Estimates (29 Imputations)							
Parameter	Estimate	Std Error	95% Confidence Limits		DF	Minimum	Maximum
INTERCEPT	-4.966510	2.326901	-9.54138	-0.39164	389.05	-6.679354	-1.400809
messupothers	0.170398	0.297150	-0.41266	0.75345	1083.1	-0.069786	0.381510
futurefoc	-0.305342	0.319975	-0.93357	0.32288	701.59	-0.579916	0.013522
healthy	0.330376	0.283618	-0.22601	0.88676	1341	0.127452	0.573156
pospeo	-3.655393	1.173725	-5.96042	-1.35037	611.3	-4.637849	-2.295354
trouble	-0.156540	0.301002	-0.74750	0.43442	709.21	-0.355970	0.171942
getsintheway	0.077028	0.351318	-0.61272	0.76678	706.5	-0.255418	0.378436
feelbad	0.137201	0.425400	-0.69824	0.97264	604.24	-0.443985	0.554698
age	0.427579	0.161211	0.11020	0.74496	271.6	0.148610	0.567156
gender_m	0.397969	0.283571	-0.15927	0.95521	465.84	0.114470	0.599268
race_cat_1	-0.098836	0.363401	-0.81513	0.61746	214.63	-0.456961	0.296123
race_cat_3	0.024178	0.334956	-0.63520	0.68356	277.25	-0.320257	0.423427
race_cat_4	0.122303	0.515733	-0.89548	1.14009	176.77	-0.543715	0.579660
lunch_yes	-0.128837	0.252038	-0.62416	0.36649	447.35	-0.393680	0.114286
baseline_vape_mon	0.077526	0.014136	0.04979	0.10526	1017.7	0.067555	0.090657
wave	-0.573755	0.194030	-0.95613	-0.19138	221.9	-0.769503	-0.234017
pospeo*wave	0.796169	0.276109	0.25335	1.33899	397.13	0.439995	1.074328

Parameter Estimates (29 Imputations)			
Parameter	Theta0	t for H0: Parameter=Theta0	Pr > |t|
INTERCEPT	0	-2.13	0.0334
messupothers	0	0.57	0.5665
futurefoc	0	-0.95	0.3403
healthy	0	1.16	0.2443
pospeo	0	-3.11	0.0019
trouble	0	-0.52	0.6032
getsintheway	0	0.22	0.8265
feelbad	0	0.32	0.7472
age	0	2.65	0.0085
gender_m	0	1.40	0.1612
race_cat_1	0	-0.27	0.7859
race_cat_3	0	0.07	0.9425
race_cat_4	0	0.24	0.8128
lunch_yes	0	-0.51	0.6095
baseline_vape_mon	0	5.48	<.0001
wave	0	-2.96	0.0034
pospeo*wave	0	2.88	0.0041

The MIANALYZE Procedure

Parameter Estimates (29 Imputations)

Parameter	*Estimate*	*Std Error*	*95% Confidence Limits*		*DF*	*Minimum*	*Maximum*
INTERCEPT	-4.952360	2.373860	-9.61745	-0.28727	454.88	-6.266429	-1.366666
messupothers	0.106865	0.300780	-0.48344	0.69717	907.53	-0.124350	0.347897
futurefoc	-0.326794	0.322755	-0.96053	0.30695	666.07	-0.620068	-0.005540
healthy	0.348762	0.287407	-0.21518	0.91270	1082.6	0.136373	0.646213
pospeo	-0.457998	0.340003	-1.12579	0.20979	577.42	-0.740424	-0.153045
trouble	-0.630196	1.017583	-2.63017	1.36978	436.57	-1.954207	0.080734
getsintheway	0.065761	0.353437	-0.62810	0.75962	740.46	-0.293201	0.366304
feelbad	0.229344	0.428604	-0.61252	1.07121	559.68	-0.370937	0.633632
age	0.379662	0.157988	0.06889	0.69043	336.42	0.118707	0.491289
gender_m	0.397260	0.288839	-0.17048	0.96500	423.28	0.072891	0.603523
race_cat_1	-0.113327	0.358398	-0.81939	0.59273	236.58	-0.517773	0.239522
race_cat_3	-0.020586	0.330440	-0.67067	0.62950	322.75	-0.329896	0.401476
race_cat_4	-0.032899	0.539161	-1.09720	1.03140	170.33	-0.682139	0.437444
lunch_yes	-0.174703	0.249950	-0.66561	0.31620	586.75	-0.393300	0.015408
baseline_vape_mon	0.078225	0.014474	0.04982	0.10663	850.32	0.069055	0.089768
wave	-0.336421	0.182553	-0.69551	0.02267	336.35	-0.527386	-0.075829
*trouble*wave*	0.107016	0.253465	-0.39157	0.60561	333.71	-0.055519	0.432268

Parameter Estimates (29 Imputations)

Parameter	*Theta0*	*t for H0: Parameter=Theta0*	*Pr > |t|*
INTERCEPT	0	-2.09	0.0375
messupothers	0	0.36	0.7225
futurefoc	0	-1.01	0.3117
healthy	0	1.21	0.2252
pospeo	0	-1.35	0.1785
trouble	0	-0.62	0.5360
getsintheway	0	0.19	0.8524
feelbad	0	0.54	0.5928
age	0	2.40	0.0168
gender_m	0	1.38	0.1697
race_cat_1	0	-0.32	0.7521
race_cat_3	0	-0.06	0.9504
race_cat_4	0	-0.06	0.9514
lunch_yes	0	-0.70	0.4849
baseline_vape_mon	0	5.40	<.0001
wave	0	-1.84	0.0662
*trouble*wave*	0	0.42	0.6731

The MIANALYZE Procedure

Parameter Estimates (29 Imputations)

Parameter	*Estimate*	*Std Error*	*95% Confidence*	*Limits*	*DF*	*Minimum*	*Maximum*
INTERCEPT	-5.167131	2.280669	-9.64929	-0.68497	447.03	-6.604338	-1.534693
messupothers	0.111083	0.299230	-0.47611	0.69827	1000.3	-0.120134	0.336515
futurefoc	-0.309444	0.319955	-0.93758	0.31870	732.15	-0.607183	0.002841
healthy	0.343461	0.286861	-0.21939	0.90631	1109.5	0.143009	0.625328
pospeo	-0.448937	0.336974	-1.11065	0.21278	637.25	-0.712264	-0.135946
trouble	-0.190321	0.304216	-0.78758	0.40694	719.97	-0.402242	0.191300
getsintheway	-1.819141	1.167781	-4.11203	0.47375	679.3	-2.577180	-0.694147
feelbad	0.209634	0.426446	-0.62797	1.04724	567.78	-0.393018	0.601947
age	0.411977	0.159338	0.09832	0.72563	279.7	0.132282	0.535920
gender_m	0.396966	0.283782	-0.16061	0.95454	490.69	0.075908	0.604100
race_cat_1	-0.107631	0.354632	-0.80605	0.59078	252.28	-0.511531	0.241732
race_cat_3	-0.003169	0.329523	-0.65155	0.64521	310.02	-0.335595	0.404344
race_cat_4	0.017019	0.523976	-1.01714	1.05117	174.29	-0.626651	0.479627
lunch_yes	-0.180280	0.247369	-0.66607	0.30551	611.91	-0.382639	0.012768
baseline_vape_mon	0.078488	0.014400	0.05023	0.10675	874.02	0.070032	0.090234
wave	-0.430958	0.182473	-0.79045	-0.07146	234.88	-0.584522	-0.098448
*getsintheway*wave*	0.473188	0.271356	-0.05969	1.00607	625.75	0.236083	0.694516

Parameter Estimates (29 Imputations)

Parameter	*Theta0*	*t for H0: Parameter=Theta0*	*Pr > |t|*
INTERCEPT	0	-2.27	0.0240
messupothers	0	0.37	0.7105
futurefoc	0	-0.97	0.3338
healthy	0	1.20	0.2314
pospeo	0	-1.33	0.1833
trouble	0	-0.63	0.5318
getsintheway	0	-1.56	0.1198
feelbad	0	0.49	0.6232
age	0	2.59	0.0102
gender_m	0	1.40	0.1625
race_cat_1	0	-0.30	0.7618
race_cat_3	0	-0.01	0.9923
race_cat_4	0	0.03	0.9741
lunch_yes	0	-0.73	0.4664
baseline_vape_mon	0	5.45	<.0001
wave	0	-2.36	0.0190
*getsintheway*wave*	0	1.74	0.0817

The MIANALYZE Procedure

Parameter Estimates (29 Imputations)							
Parameter	*Estimate*	*Std Error*	*95% Confidence*	*Limits*	*DF*	*Minimum*	*Maximum*
INTERCEPT	-5.327579	2.361794	-9.96739	-0.68777	520.82	-7.163149	-1.697966
messupothers	0.115082	0.301483	-0.47656	0.70672	963.5	-0.118543	0.362265
futurefoc	-0.342417	0.322233	-0.97495	0.29012	787.68	-0.620266	-0.021696
healthy	0.341267	0.289880	-0.22752	0.91006	1081.9	0.127576	0.634900
pospeo	-0.463859	0.339601	-1.13071	0.20300	646.16	-0.741947	-0.182630
trouble	-0.211514	0.307403	-0.81503	0.39200	716.86	-0.427747	0.183881
getsintheway	0.055555	0.356359	-0.64403	0.75514	746.14	-0.290739	0.340108
feelbad	0.809552	1.352587	-1.84944	3.46854	404.02	-1.232643	2.162224
age	0.387997	0.160738	0.07183	0.70416	339.91	0.122756	0.521995
gender_m	0.393367	0.287557	-0.17165	0.95838	483.41	0.083276	0.578684
race_cat_1	-0.113089	0.360623	-0.82336	0.59719	248.02	-0.511676	0.245563
race_cat_3	-0.008221	0.332278	-0.66184	0.64540	334.65	-0.317414	0.423396
race_cat_4	0.018374	0.529523	-1.02621	1.06295	187.74	-0.607393	0.476973
lunch_yes	-0.188277	0.253753	-0.68674	0.31018	541.39	-0.414230	0.036992
baseline_vape_mon	0.078658	0.014508	0.05018	0.10713	814.23	0.070213	0.091551
wave	-0.273945	0.165334	-0.59930	0.05141	301.02	-0.427529	-0.010317
*feelbad*wave*	-0.149756	0.347969	-0.83490	0.53538	264.68	-0.515401	0.413062

Parameter Estimates (29 Imputations)			
Parameter	*Theta0*	*t for H0: Parameter=Theta0*	*Pr > |t|*
INTERCEPT	0	-2.26	0.0245
messupothers	0	0.38	0.7028
futurefoc	0	-1.06	0.2883
healthy	0	1.18	0.2393
pospeo	0	-1.37	0.1724
trouble	0	-0.69	0.4916
getsintheway	0	0.16	0.8762
feelbad	0	0.60	0.5498
age	0	2.41	0.0163
gender_m	0	1.37	0.1720
race_cat_1	0	-0.31	0.7541
race_cat_3	0	-0.02	0.9803
race_cat_4	0	0.03	0.9724
lunch_yes	0	-0.74	0.4584
baseline_vape_mon	0	5.42	<.0001
wave	0	-1.66	0.0986
*feelbad*wave*	0	-0.43	0.6673
